# High electron transfer efficiency accordion-shaped HNiZn heterostructure nanozyme for low-temperature photo-catalytic enhanced therapy of bacterial infection wounds

**DOI:** 10.1016/j.mtbio.2025.102097

**Published:** 2025-07-16

**Authors:** Hanjie Wang, Xinqi Guo, Ying Tan, Junxu Yang, Yuting Ye, Miao Mo, Yanling Liang, Guanhua Li, Zhangrui Huang, Li Zheng, Xiaofei Ding, Jingping Zhong, Jinmin Zhao

**Affiliations:** a Guangxi Engineering Center in Biomedical Material for Tissue and Organ Regeneration, Collaborative Innovation Centre of Regenerative Medicine and Medical BioResource Development and Application Coconstructed By the Province and Ministry, Guangxi Key Laboratory of Regenerative Medicine, The First Affiliated Hospital of Guangxi Medical University, No. 6 Shuangyong Road, Nanning, Guangxi, 530021, PR China; b Life Sciences Institute, Guangxi Medical University, No. 22 Shuangyong Road, Nanning, Guangxi, 530021, PR China; c Department of Orthopaedics Trauma and Hand Surgery, The First Affiliated Hospital of Guangxi Medical University, No. 6 Shuangyong Road, Nanning, Guangxi, 530021, PR China

**Keywords:** Nanozymes, Heterojunction, Bacterial infection, Reactive oxygen species, Photothermal therapy

## Abstract

Bacterial and drug-resistant bacterial infections pose significant challenges to the treatment of skin wounds. Among various non-antibiotic strategies, nanozymes which mimic the activities of natural bioenzymes and possess broad-spectrum antibacterial properties, hold promise for antibacterial therapy in infected wounds. However, the catalytic activity and biosafety of most current nanozymes remain insufficient to meet clinical requirements. Herein, we innovatively synthesized novel heterostructured nanozymes (HNiZn) comprising Ni_4_N/Ni_3_ZnC_0.7_ embedded in accordion-shaped nitrogen-doped carbon using a simple molten-salt pyrolysis method. Combined with injectable hyaluronic acid (HA) as a carrier, these nanozymes facilitate low-temperature (43.5 °C) photocatalytic and photothermal therapy for bacterially infected wounds. Based on density functional theory (DFT) calculations, the Ni_4_N/Ni_3_ZnC_0_._7_ heterostructured nanozymes exhibit richer electron cloud distribution, stronger interactions between heterogeneous atoms, lower electron escape work function, stronger adsorption energy for free radicals, and electron transfer efficiency than individual Ni_4_N or Ni_3_ZnC_0.7_ phases, resulting in efficient peroxidase (POD)-like and glutathione peroxidase (GPx)-like activities. Additionally, HNiZn exhibits a high photothermal conversion efficiency (51.01 %) under near infrared (NIR) irradiation. Through combined photocatalytic and photothermal effects, it effectively kills *Escherichia coli* (*E. coli*), clinically isolated methicillin-resistant *Staphylococcus aureus* (MRSA), and their biofilms. Mechanistic studies using metabolomics analysis revealed that HNiZn induces bacterial apoptosis by disrupting bacterial biosynthesis and metabolism, affecting the cell cycle, and perturbing redox balance. In vivo experiments further confirmed the favorable biosafety and antibacterial efficacy of HNiZn, which promoted skin wound healing. This study provides a novel strategy for constructing effective nanozymes and treating bacterial infections.

## Highlights

• Innovative synthesis of hyaluronic acid-modified accordion-like heterojunction HNiZn for bacterial-infection wound treatment.
• HNiZn has a high PCE (51.01 %) and can perform photocatalysis and photothermal therapy under low-temperature PTT conditions.
• Ni_4_N/Ni_3_ZnC_0.7_ heterostructure has higher electron transfer efficiency; HNiZn shows superior POD-like and Gpx-like activities.
• HNiZn effectively kills E. coli and MRSA, promotes the healing of MRSA-infected wounds, and exhibits biosafety.
• Mechanistically, HNiZn induces apoptosis by interfering with bacterial biosynthesis and disrupting bacterial redox balance. Please confirm if these meet the requirements.

## Graphical abstract

**Figure:**
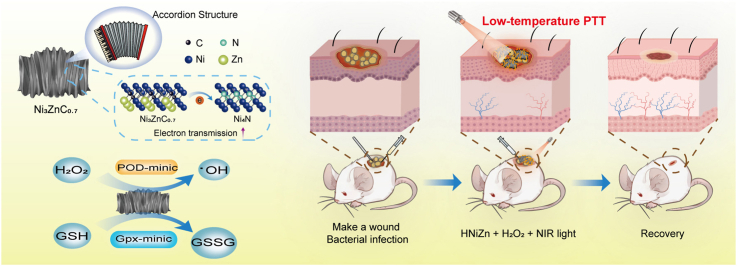

## 1 Introduction

Bacterial infections, particularly those involving drug-resistant strains, present significant challenges to the treatment of skin wounds. These infections remain an important public health concern, adversely affecting patient health [1,2]. Antibiotics are commonly used to treat bacterial infections. However, antibiotic misuse has contributed to the emergence of drug-resistant bacteria that develop tolerance to high antibiotic doses and necessitate prolonged treatment duration [3,4]. Furthermore, Long-term antibiotic use carries risks such as organ toxicity and increased drug resistance, ultimately compromising wound healing [5].

In recent years, nanozymes have been widely used in therapeutic antibacterial research, due to their high efficiency, stability, ease of surface functionalization, low cost, and convenient storage [6,7]. Among them, heterostructure nanozymes are composed of two or more different materials (such as semiconductors, metals, oxides, sulfides, etc.), forming crystal structures at the interface [8]. This interfacial effect can generate electron holes, enrich interfacial defects, and enhance the exposure of the active site, thereby enhancing the enzyme-like activities such as peroxidase (POD) and glutathione peroxidase (GPx). Therefore, it has broad-spectrum antibacterial activity, low drug resistance, and high stability, and has great potential in the treatment of infectious diseases [9,10]. For example, Wang et al. synthesized defect-rich ruthenium oxide/iridium oxide (RuO_2_/IrO_2_) heterojunction nanosheets via a molten salt method. These nanosheets exhibited significantly enhanced biocatalytic activity, leading to excellent antimicrobial properties [11]. Similarly, Wu et al. developed an antibiotic-free Schottky heterojunction (Ag_2_S/Ti_3_C_2_) for bacterial infection treatment, achieving 99.99 % antimicrobial efficiency through the combined photocatalytic and photothermal effects [12]. However, the existing heterojunction nanozymes still face the problems of complicated preparation process, high charge complexity, and difficult interfacial electron regulation, leading to compromised catalytic activity and limited antibacterial potential [13,14]. Notably, the introduction of the transition metal nitrides (TMNs) and carbides (TMCs) addresses these deficiencies due to their high electrical conductivity, excellent chemical stability, and catalytic activity [15,16]. For example, Yang et al. employed a cation exchange reaction (CER) with Cu_3_N as a template to synthesize Ni_4_N/CoN core-shell heterojunctions in a single step. The strong electronegativity of nitrogen shifted the delectron state density of Co closer to the Fermi level, optimizing the adsorption energy and electronic structure of the oxygen intermediates, thereby lowering the reaction energy barrier. Moreover, CoN exhibited high electron transport efficiency, which significantly enhances the reaction kinetics [17]. However, challenges remain, such as balancing biosafety with catalytic activity, auncontrollable synthesis, and unclear structure-activity relationship [18].

The multi-enzyme catalysis of nanozymes combined with photothermal therapy (PTT) to achieve synergistic antimicrobial effects has emerged as a promising strategy [[19], [20], [21]]. For example, Feng et al. developed Au/Cu nanoparticles confined on lysozyme amyloid nanofiber mesh, with a photothermal conversion efficiency of 25.1 %, which can effectively kill bacteria under high temperature (48 °C) near-infrared II (NIRII, 808 nm) irradiation [22]. And Bai et al. reported 2D porous N-referenced nanosheets loaded with CuNx nanosystems, which effectively inhibited multi-drug-resistant bacteria under NIRII irradiation (with a photothermal conversion efficiency of 40.9 %, 50 °C) [23]. Thus, the PTT effect not only enhanced enzyme activity, but also reduced cytotoxicity by lowering the drug dosage for skin wound healing, thus meeting the clinical needs for efficient and safe sterilization. However, excessively high temperatures may also cause damage to the human body [24]. Therefore, our study aims to design of a heterostructure nanozyme, which utilized its efficient multi-enzyme activity in combination with low-temperature PTT (<45 °C), thereby killing drug-resistant bacteria safely and efficiently.

**Scheme 1:**
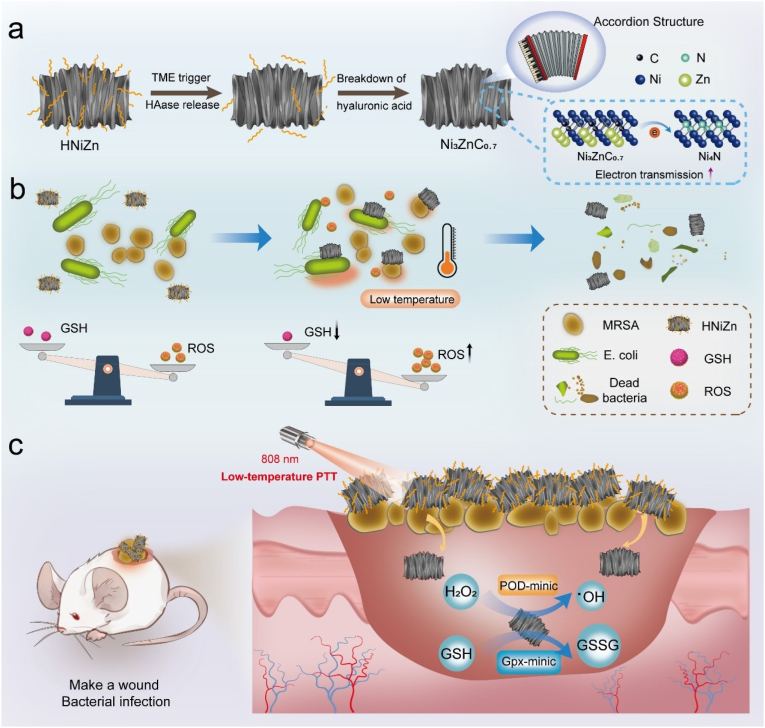
(a) Schematic diagram of HNiZn exerting enzyme-like activity in the breakdown of hyaluronic acid in the infected microenvironment. (b) Schematic diagram of HNiZn mimicking POD enzyme and Gpx enzyme to eradicate *E. coli* and MRSA in vitro. (c) HNiZn was used to treat MRSA infection in vivo.

Herein, in this work, a unique heterostructured nanozyme was innovatively synthesized by embedding Ni_4_N/Ni_3_ZnC_0.7_ in accordion-like nitrogen-doped carbon (HNiZn) through a simple molten-salt pyrolysis method, with hyaluronic acid (HA) serving as a vehicle, for photocatalytic and PTT of bacterial-infected wounds under low-temperature (43.5 °C) PTT (Scheme 1). Results showed that HNiZn exhibited high photothermal conversion efficiency (51.01 %), and the electron holes generated by its accordion-shaped heterostructure, rich electron cloud distribution, strong interactions between heterogeneous atoms, low electron escape work, and strong adsorption energy for free radicals, effectively promote electron transfer, endowing it with excellent POD-like activity to catalyze the production of ·OH from hydrogen peroxide and GPx-like activity to consume GSH, thus effectively killing *Escherichia coli* (*E. coli*) and clinically isolated methicillin-resistant *Staphylococcus aureus* (MRSA). Moreover, HNiZn effectively inhibited biofilm formation and exhibited favorable eradication activity against clinically isolated MRSA under NIR irradiation. Importantly, HNiZn effectively promoted the healing of MRSA-infected wounds and was proven to be biosafe in vivo. Mechanistically, HNiZn induced apoptosis by affecting carbonic anhydrase required for bacterial reproduction, interfering with bacterial biosynthesis, nitrogen and energy metabolism, inducing cell cycle arrest, and disrupting bacterial redox balance. Therefore, this study expands the application of heterogeneously structured nanozymes in diseases associated with bacterial infections.

## 2 Materials and methods

### 2.1 Materials

Sinopharm Chemical Reagent Co. provided the sodium chloride (NaCl), zinc nitrate hexahydrate (Zn(NO_3_)_2_, 6H_2_O), and nickel nitrate hexahydrate (Ni(NO_3_)_2_, 6H_2_O). Aladdin supplied the melamine. The supplier of the cell counting kit (CCK-8) was BioSharp Brand. Sodium hyaluronate was provided by Shanghai Yuanye. MRSA (Mu50) and *E. coli* (HB101(RP4)) standard strains were from Ru Microbial Technology Co. O11 probe and N01/PI staining solution were from Shanghai Beibao Biotechnology Co. Crystalline violet staining solution was from Biyuntian Biotechnology Co. HE staining and Masson staining kits were provided by Soleberg. VEGF, α-SMA and CD31 antibodies were provided by PhD Bioengineering Co.

### 2.2 Synthesis of Ni_4_N/Ni_3_ZnC_0.7_ and HNiZn

0.2 g of Zn(NO_3_)_2_, 6H_2_O, 0.1 g of Ni(NO_3_)_2_, 6H_2_O, and 0.8 g of NaCl were combined and crushed continually to produce a light green powder. Next, the resulting light green powder was heated with 1.0 g of melamine at 500 °C for 2 h under an Ar gas atmosphere, then pyrolyzed at 800 °C for 2 h at a ramping rate of 2 °C per minute, and subsequently rinsed at room temperature to extract Ni_4_N/Ni_3_ZnC_0.7_ by removing any remaining sodium chloride. 20 mL of ultrapure water were mixed with 10 mg of the product and 5 mg of sodium hyaluronate. The product was stirred for 12 h, then rinsed three times with ultrapure water and placed in a lyophilizer for 48 h of continuous lyophilization. HNiZn was obtained as the final product. Next, the resulting light green powder was heated with 1.0 g of melamine at 500 °C for 2 h under Ar gas atmosphere, then pyrolyzed at 800 °C for 2 h at a ramping rate of 2 °C per minute, and then rinsed at room temperature to extract Ni_4_N/Ni_3_ZnC_0.7_ by eliminating any remaining sodium chloride.

### 2.3 Characterization

Using field emission scanning electron microscopy (FE-SEM, Phenom Pharos), scanning electron microscopy imaging. Using a field-emission electron microscope (JEM-2100F, Japan) with an EDS detector (Oxford Instrument, UK), TEM imaging and elemental analysis were carried out. AFM (MFP-3D system) was used to analyze the morphology and microstructure of the samples, and the LabRAM HR Evolution micro-Raman Spectrometer (HORIBA Jobin Yvon, France) with a laser spot diameter of approximately 600 nm and an excitation wavelength of 532 nm was used to characterize the crystal structure. Using a Kubo-X1000 surface analyzer, the pore structures of the catalysts were examined as they were synthesized. Using an X-ray diffractometer (Rigaku, Ultima IV, Japan), the XRD spectrum was examined. We used the Thermo Fisher ESCALAB 250Xi, USA, for XPS analysis. Images of the cells obtained using a confocal laser scanning microscope (CLSM) were viewed using a CLSM (Leica Application Suite X). An FLIR C2 NIR thermal imaging camera was used to provide photothermal pictures both in vitro and in vivo. Section staining relevant to immunohistochemistry was examined with a florescent inverted microscope.

### 2.4 Photothermal properties of HNiZn

A 1.5 mL Eppendorf tube was filled with 1 mL of HNiZn solution (0, 10, 20, 30, 40, 50, 100 μg mL^−1^) or ultrapure water.The tube was subsequently exposed to radiation for 10 min (808 nm, 1.0 W cm^−2^), and the temperature of the mixture was recorded every 30 s. A 1.5 mL Eppendorf tube was filled with 1 mL of HNiZn solution (50 μg mL^−1^) and exposed to varying powers (808 nm, 0.5, 0.75, 1.0, 1.5 W cm^−2^) for 10 min. The temperature of the solution was recorded every 30 s to examine the effect of the varying powers on the photothermal properties. After three warming and cooling cycles, the photothermal stability of HNiZn was evaluated.

### 2.5 Peroxidase activity of HNiZn

The POD-like activity of HNiZn was measured in the presence of H_2_O_2_, using TMB as a substrate. TMB is a naturally occurring substrate for POD, which can be oxidized by the enzyme to produce oxTMB, characterized by a distinctive blue hue and an absorption wavelength of 652 nm. After the addition of HNiZn (0, 10, 20, 30, 40, and 50 μg mL-^1^), H_2_O_2_ (0.1 M), and TMB (10 mM) to the cuvette, 3 mL of a pH 4.5 sodium acetate solution was introduced, and the color change of the solution, along with its UV–Vis–NIR spectra, was observed. Additionally, HNiZn (50 μg mL^−1^), H_2_O_2_ (0.1 M), and TMB (10 mM) were reacted at various times and in buffer solutions of different pH (pH 4.5, 5.5, 6.5, and 7.5) to explore the pH and time dependence of POD-like activity. Changes in the peak absorbance at 652 nm were detected.

### 2.6 ESR for ·OH detection

The hydroxyl radical derived from H_2_O_2_ solution, for 5 min, was produced under the irradiation with a full-band xenon lamp and captured by 5-tert-butoxycarbonyl 5-methyl-1-pyrroline-N-oxide (BMPO, 10 mM). When hydroxyl radicals were generated, four peaks on the ESR spectrometer were observed. The ability of HNiZn nanozyme to produce ·OH at different concentrations was evaluated by observing the change in peak intensity.

### 2.7 Glutathione peroxidase activity of HNiZn

GSH depletion was revealed using a DTNB probe and UV–Vis–NIR spectroscopy. Glutathione (1.0 mM) was combined with the resultant HNiZn at various concentrations (10, 20, 30, 40, and 50 μg mL^−1^) at room temperature. 900 μL of saline solution containing phosphate buffer (PBS, pH 7.4) was mixed with 100 μL of the mixture at various intervals. DTNB (0.1 mM) was subsequently added, and the UV–Vis–NIR spectra were recorded.

### 2.8 Enzyme kinetic analysis

Kinetic experiments were performed by varying the concentrations of H_2_O_2_ and GSH and were calculated by a typical Michaelis-Menten curve. For H_2_O_2_, the mixed solution contains 50 mg mL^−1^ HNiZn, 10 mM TMB, and 0–1 mM H_2_O_2_; For GSH, the mixed solution contains 50 mg mL^−1^ HNiZn, 0.1 mM of DTNB, and 0–6 mM GSH.

### 2.9 Density functional theory (DFT) Computational details

All DFT calculations in this study are performed by with the Vienna ab initio simulation package (VASP) [25]. The Perdew-Burke-Ernzerhof (PBE) [26] functional was employed to treat the exchange-correlation interactions. The plane-wave basis set with a kinetic energy cutoff of 400 eV, the energy convergence criterion of 10^−4^ eV, the force convergence criterion of 0.02 eV Å^−1^, and a (1 × 1 × 1) Monkhorst-Pack k-point sampling were employed for structure relaxation. For different surface models, the bottom layer is fixed. A sufficiently large vacuum gap (>12 Å) was employed to prevent the interaction between neighboring periodic structures along the c-axis. H_2_O_2_ was calculated in boxes of 20 Å × 20 Å × 20 Å with the gamma point only. The adsorption energy (Eads) of the adsorbate H_2_O_2_ was defined as

$$E_{\text{ads}}=E_{\text{sur}+H2O2}-E_{\text{sur}}\;–\;E_{H2O2}$$

where E_sur + H__2__O2_, E_sur_ and E_H2O2_ represent the total energy of slab after adsorption with H_2_O_2_, the energy of the bare slab and the energy of the H_2_O_2_, respectively.

The work function was defined as

$$\Phi =E_{\text{vac}}-E_{f-M}$$

where E_vac_ and E_f-M_ represent the Fermi level and the vacuum level.

Ni_4_N/Ni_3_ZnC_0.7_, Ni_4_N and Ni_3_ZnC_0.7_ nanozymes were used to study the superoxide POD-like activity comparatively.

### 2.10 In vitro antimicrobial assay

The antimicrobial investigation utilized methicillin-resistant Staphylococcus aureus (MRSA, Mu50) as a Gram-positive bacterial model and *Escherichia coli* (*E. coli*, HB101 (RP4)) as a Gram-negative bacterial model. The bacterial density was determined by measuring the absorbance at 600 nm. In a typical antimicrobial test, MRSA or *E. Coli* were divided into eight groups:(1) Bacteria; (2) Bacteria + NIR; (3) Bacteria + H_2_O_2_; (4) Bacteria + H_2_O_2_+NIR; (5) Bacteria + HNiZn; (6) Bacteria + HNiZn+NIR; (7) Bacteria + HNiZn+H_2_O_2_; (8) Bacteria + HNiZn+H_2_O_2_+NIR After NIR irradiation, groups (2), (4), (6), and (8) were exposed to NIR laser (808 nm, 1.0 W cm^−2^) for 10 min. The protocol remained consistent from groups (1), (3), (5), and (7). The final concentrations of H_2_O_2_, HNiZn, and bacteria were 100 μM mL^−1^, 50 μg mL^−1^, and approximately 1 × 10^8^ CFU mL^−1^, respectively. One milliliter (mL) of solution was added to each well. The bacterial suspensions from groups (1) to (8) were collected and evenly spread on agar plates after 4 h of incubation. Colonies were counted after the plates were incubated for 24 h at 37 °C. Each experiment was performed in triplicate. The bacterial survival rate was determined using the following formula: survival viability (%) = (Nt/Nc) × 100 %, where Nt and Nc represent the number of colonies in the experimental and control (PBS) groups, respectively.

### 2.11 Live/dead staining experiment

After various treatments, the bacteria were stained with N01 and PI for 15 min. The bacteria were subsequently rinsed once with 0.85 % NaCl to remove any remaining external dyes, and the living (green fluorescence) and dead (red fluorescence) bacteria were observed using a fluorescence microscope.

### 2.12 ROS staining assay

The O11 (Ros fluorescence) probe was employed to investigate the production of ROS by bacteria. After various treatments, the bacteria were rinsed with PBS following incubated with the O11 probe at 37 °C for 45 min, under light protection. ROS production was confirmed by fluorescence microscopy, which revealed green fluorescence.

### 2.13 Bacterial SEM preparation process

After various treatments, first, gently centrifuge the bacteria 2–3 times with buffer (such as 0.1M PBS or normal saline), remove the culture medium residue, and adjust the concentration of bacteria suspension to 10^6^∼10^8^ CFU mL^−1^. Next, the cells were fixed at 4 °C for 2–4 h using 2.5 % glutaraldehyde, and then rinsed with PBS for 10 min each time to completely remove glutaraldehyde. Subsequently, 1 % osmic acid was used to fix it at room temperature for 1–2 h to enhance the conductivity and contrast of the sample, and wash it with PBS or deionized water three times again. Next, ethanol gradient dehydration is used, soak 30 %, 50 %, 70 %, 80 %, 90 %, 100 % ethanol step by step. Each stage is 10∼15 min, and 100 % ethanol twice to ensure complete dehydration. After that, hexamethyldisilazane (HMDS) was used for drying, and after treatment with 100 % ethanol, HMDS (1:1), and pure HMDS, it was placed in air to evaporate and dry. Finally, the dried samples are adhered to the sample table with conductive glue or carbon tape, and ion sputtering coating is performed, and the 5–10 nm gold/platinum layer is sprayed (common parameters are 10–20 mA, 60–120 s).

### 2.14 Biofilm culture experiment

Single colonies of MRSA were inoculated into 100 mL LB broth and cultured at 37 °C until the bacterial suspension reached the log phase. Then, 10 μL of bacterial suspension was adjusted to the final concentration of OD600 nm = 0.01, and a sterile confocal Petri dish containing 1 mL of TSB medium (0.25 wt% glucose) and a sterile steel dish was added to the biofilm growth. The plates were covered and sealed with a sterile conservative membrane and incubated statically at 37 °C, replacing the medium every 24 h.

### 2.15 Anti-bacterial biofilm assay

24-well plates containing a suspension of methicillin-resistant MRSA at a concentration of 1 × 10^8^ CFU mL^−1^ were incubated for 48 h at 37 °C. Eight experimental groups were established: (1) Bacteria; (2) Bacteria + NIR; (3) Bacteria + H_2_O_2_; (4) Bacteria + H_2_O_2_+NIR; and (5) Bacteria + HNiZn. Each well was exposed to radiation for 10 min, with or without an 808 nm laser (1.0 W cm^−2^) under the following conditions: (6) Bacteria + HNiZn+NIR; (7) Bacteria + HNiZn + H_2_O_2_; (8) Bacteria + HNiZn + H_2_O_2_+NIR. Crystalline violet (0.1 %) was subsequently added to each well, and each well was photographed using a digital camera for 10 min. Following the addition of 500 μL of ethanol to each well, the biomass of the biofilm was quantified by measuring the absorbance of each sample at 590 nm.

### 2.16 Morphological study of biofilm

There were still eight experimental groups created: (1) Bacteria; (2) Bacteria + NIR; (3) Bacteria + H_2_O_2_; (4) Bacteria + H_2_O_2_+NIR; and (5) Bacteria + HNiZn. Each well was exposed to radiation for 10 min either with or without an 808 nm laser (1.0 W cm^−2^) in the following conditions: (6) Bacteria + HNiZn+NIR; (7) Bacteria + HNiZn + H_2_O_2_; (8) Bacteria + HNiZn + H_2_O_2_+NIR. Mature MRSA biofilm samples were cultured in isotonic saline with the nanosystems described above to fully identify the biofilm matrix, and samples were carefully washed several times with isotonic saline to remove randomly attached nanozymes for further CLSM analysis with SYTO-9/PI staining.

### 2.17 Metabolite analysis experiments

Bacterial samples were centrifuged after different treatments. The supernatants were removed, followed by metabolite extraction. An LS-MS/MS assay was performed, differential metabolites were identified and screened, and bioinformatics analysis was conducted.

### 2.18 In vivo experiments

The Animal Care & Welfare Committee of Guangxi Medical University (approved protocol number: 202206158) were followed in conducting all animal research. Six groups (n = 5) consisting of SD rats were randomly assigned: (1) Control group; (2) H_2_O_2_ group; (3) HNiZn; (4) HNiZn+NIR group; (5) HNiZn + H_2_O_2_ group; and (6) HNiZn + H_2_O_2_+NIR group. These SD rats had their backs shaved, lithium pentobarbital was used to induce unconsciousness, and a sterile scalpel was used to make a 2 cm^2^ incision in the skin. Subsequently, the wound was gradually infected with S. aureus suspension (50 μL, 1 × 10^8^ CFU mL^−1^), and the moisture was allowed to evaporate spontaneously. A day later, group (6) received an inoculation containing 50 μL HNiZn (50 μg mL ^−1^) and 50 μL of H_2_O_2_ (100 μM) applied to the infected lesion. Following this, the wound was exposed to a 5-min exposure using a near-infrared laser (808 nm, 1.0 W cm^−2^). The experiment was conducted with the remaining five groups with the identical protocol, whereas the control group alone utilized phosphate buffer. Following the various treatments, the rats' body weight was noted, and picture g was used to calculate the trauma size of each set of rats. Rat skin specimens were collected for histopathological hematoxylin-eosin (HE), Masson's trichrome, and immunohistochemical staining investigation of VEGF and α-SMA following a seven-day trauma area therapy period.

### 2.19 In vivo biosafety

Following the execution of each set of rats, blood was drawn for standard and biochemical tests, and the primary organs (heart, liver, spleen, lungs, and kidneys) were stained with HE staining to assess the biosafety of HNiZn.

### 2.20 CCK-8 experiment

Rat fibroblasts confirmed that HNiZn was cytocompatible. First, the cells were cultivated for 24 h in 96-well plates after being injected. The cells were subsequently washed twice or three times with PBS, treated with CCK-8 solution for 2 h, and then exposed to several doses (0, 5, 10, 20, 30, 50, 80, and 100 μg mL^−1^) of Ni_4_N/Ni_3_ZnC_0.7_ and HNiZn for a whole day. The microtiter plate device was used to measure the absorbance (OD) at 450 nm. At 450 nm, the absorbance (OD) was measured.

### 2.21 Live/dead cell staining assay

HUVECs at a density of 10^5^ cells per well were cultured in 6-well plates for 24 h to allow cell attachment. After washing the cells twice with PBS solution, the cells were divided into two groups: control and HNiZn group. After treatment, the cells were stained with calcein AM and PI for 20 min and finally observed by fluorescence microscopy.

### 2.22 Hemolysis experiment

The hemolysis rate of blood cells incubated with varied HNiZn amounts was used to assess its hemocompatibility. SD rat whole blood was spun up with 1000 rpm for 10 min to extract blood cells precipitates. Centrifugation revealed the whitish supernatant after repeated PBS washings of precipitated erythrocytes. To create the ultimate concentrations of 20, 40, 60, 80, and 100 μg mL^−1^, 0.5 mL of deionized water (positive control) and various HNiZn concentrations were mixed into 0.5 mL of 4 % erythrocytes. After 4 h of incubation at room temperature, the supernatants were spun up and an enzyme marker at 540 nm measured absorbance. Hemolysis rate (%) = A/A_1_ × 100 %, where A and A_1_ represent HNiZn and the deionized water leftovers absorbances.

### 2.23 Calculation of the photothermal conversion efficiency

According to Roper's report, the total energy balance of the system input and dissipation can be expressed as

$$\eta =\frac{\text{hs}\left(T_{\max}‐T_{\text{surr}}\right)‐Q_{\text{dis}}}{I‐I\times 10^{‐A808\text{nm}}}$$

Tmax refers to the maximum temperature at the time of illumination, Tsurr refers to the ambient temperature at the time of illumination, I refer to the power density, and A refers to the UV absorption at 808 nm of the material used for illumination.

$$Q_{\text{dis}}=\frac{\text{mc}\Delta T}{t}$$

Qdis the energy released by the absorption of light by an equal amount of pure water solution, m refers to the mass of water, c refers to the specific heat capacity of water (4.2 × 10^3^ J), ΔT is the value of the temperature drop of the pure water solution after turning off the laser, and t is the cooling time of pure water.

$$\text{hS}=\frac{\text{mcln}\theta }{t}$$

h is the heat transfer coefficient, S is the surface area of the vessel, t is the time.

$$\theta =\frac{T-T_{0}}{T_{\max}-T_{0}}$$

T is the temperature of the solution at different moments in the natural cooling state, T_max_ is the maximum temperature that can be reached in the equilibrium state of the solution under laser irradiation, and T_0_ is the starting temperature of the solution.

### 2.24 Statistical analysis

The statistical software SPSS was utilized for data analysis, and the outcomes were presented as mean ± standard deviation (SD). GraphPad Prism 8.0.1 (USA) was used to create the graphs. A one-way ANOVA was used to establish statistical significance. It was deemed statistically significant when P < 0.05.

## 3 Results and discussion

### 3.1 Preparation and characterization of Ni_4_N/Ni_3_ZnC_0.7_ and HNiZn

**Fig. 1:**
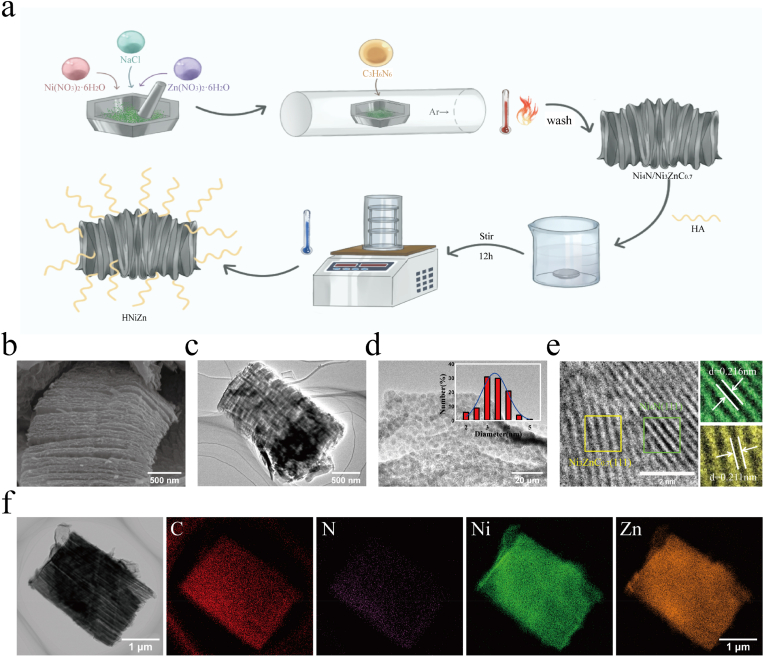
(a) Schematic of the synthesis of Ni_4_N/Ni_3_ZnC_0.7_ and HNiZn. (b) SEM images of Ni_4_N/Ni_3_ZnC_0.7_. (c) TEM images of Ni_4_N/Ni_3_ZnC_0.7_. (d) High-resolution TEM (HR-TEM) images of Ni_4_N/Ni_3_ZnC_0.7,_ along with the corresponding nanoparticle size distribution histogram (inset), are presented. (e) HR-TEM image of Ni_4_N/Ni_3_ZnC_0.7_. (f) EDX elemental mapping of Ni_4_N/Ni_3_ZnC_0.7_.

**Fig. 2:**
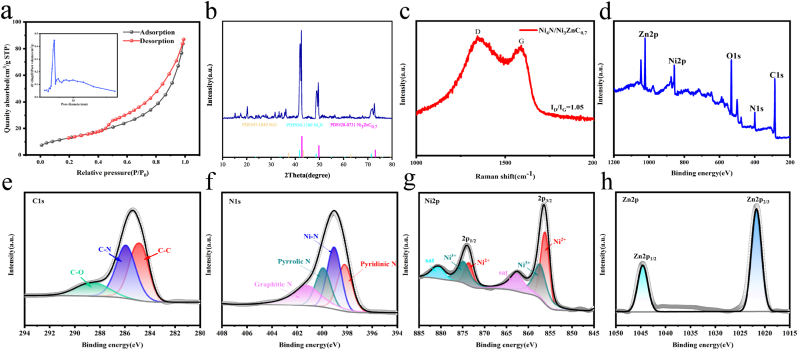
(a) Nitrogen isothermal adsorption and desorption curves. (b) XRD spectra. (c) Raman spectra. (d) XPS and high-resolution XPS spectra of (e)C 1s, (f)N 1s, (j)Ni 2p, (h)Zn2p.

The Ni_4_N/Ni_3_ZnC_0.7_ heterojunction was synthesized using a straightforward molten salt annealing method, followed by the addition of hyaluronic acid (HA) to form HNiZn nanomaterials (Fig. 1a). Scanning electron microscopy (SEM) was used to examine the morphology of the manufactured samples, revealing that the Ni_4_N/Ni_3_ZnC_0.7_ heterojunction exhibits a uniform layered structure (Fig. 1b). The surface of the Ni_4_N/Ni_3_ZnC_0.7_ heterojunction was evenly wrapped following HA treatment (Fig. s1). Transmission electron microscopy (TEM) images revealed that the lamellar structure of Ni_4_N/Ni_3_ZnC_0.7_ resembles an accordion and consists of several vertically oriented nanosheets (Fig. 1c). High-resolution transmission electron microscopy (HR-TEM) images clearly showed uniform dispersion of Ni_4_N/Ni_3_ZnC_0.7_ nanoparticles. As shown in the inset of Fig. 1d, the average particle size is approximately 3.353 ± 0.606 nm, with lattice spacings of 0.216 nm and 0.211 nm, corresponding to the (111) crystal planes of Ni_4_N and Ni_3_ZnC_0.7_(Fig. 1e), respectively. This layered mesoporous structure facilitates the adsorption of chemical intermediates that resemble enzymes and exposure many active sites, both of which contribute to enhanced catalytic activity. It also simplifies the construction of channels for efficient transport. Fig. 1f showed that the energy-dispersive X-ray (EDX) elemental mapping images confirm the homogeneous distribution of C, N, Ni, and Zn across the heterojunction, with a Ni/Zn atomic ratio of approximately 1:1 (Fig. s2 and Table s1). In addition, as shown in Figures s3 and s4a-d, HA was successfully modified onto the surface of the Ni_4_N/Ni_3_ZnC_0.7_ heterojunction. The specific surface area and pore structure of Ni_4_N/Ni_3_ZnC_0.7_ were assessed using adsorption-desorption isotherms (Fig. 2a). Ni_4_N/Ni_3_ZnC_0.7_ exhibited a specific surface area of 48.7 m^2^ g^−1^ and a uniform size distribution. The large specific surface area of the material may have facilitated substrate diffusion, which was advantageous for exposure of enzyme-triggered reaction sites. Similarly, the cubic Ni_3_ZnC_0.7_ (PDF#28–0713) exhibited three crystallographic diffraction peaks at 42.5, 49.5, and 72.7 on the X-ray diffraction (XRD) pattern of Ni_4_N/Ni_3_ZnC_0.7_, as shown in Fig. 2b. These peaks correspond to the (111), (200), and (220) crystallographic planes. The distinctive peaks of Ni_4_N (PDF#36–1300) include three additional diffraction peaks at 42.0, 48.9, and 71.9. Both Ni_4_N and Ni_3_ZnC_0.7_ possess a cubic crystal structure with a well-matched lattice, enabling the formation of an efficient heterojunction. Furthermore, in the Raman spectrum of Ni_4_N/Ni_3_ZnC_0.7_ (Fig. 2c), two peaks were detected near 1340 and 1580 cm, corresponding to the sp^2^ hybridized D-band and sp^3^ hybridized G-band of carbon, respectively, with an *I*_*D*_*/I*_*G*_ ratio of 1.05. This implying that more defects were generated in Ni_4_N/Ni_3_ZnC_0.7_, which was beneficial for free radical adsorption and enzyme electron transfer during catalyzed reactions.

The electrical structure of Ni_4_N/Ni_3_ZnC_0.7_ was examined using X-ray photoelectron spectroscopy (XPS). The Ni_4_N/Ni_3_ZnC_0.7_ heterojunction (Fig. 2d) exhibits the presence of C, N, Ni, Zn, and O across the entire XPS spectrum. Three peaks at 284.8, 285.9, and 288.4 eV, which correspond to C-C, C-N, and C-O, respectively, were fitted to the high-resolution spectrum of C1s of Ni_4_N/Ni_3_ZnC_0.7_ (Fig. 2e), indicating nitrogen doping in carbon nanosheets. Furthermore, pyridine N (398.2 eV), Ni-Nx (399.0 eV), pyrrole N (399.9 eV), and graphite N (401.2 eV) were all be matched to the high-resolution spectra of N1s (Fig. 2f). Ni^2+^ (856.1 eVp3/2 and 873.5 eV2p1/2) and Ni^3+^ (857.3 eVp3/2 and 874.7 eV2p1/2) represent the two pairs of peaks assigned to Ni^2+^ in the Ni2p spectra of Ni_4_N/Ni_3_ZnC_0.7_, along with satellite peaks at 862.6 and 880.5 eV (Fig. 2g). Additionally, two distinct peaks were observed in the Zn2p high-resolution XPS spectra of Ni_4_N/Ni_3_ZnC_0.7_: Zn2p3/2 at 1022.4 eV and Zn2p1/2 at 1045.4 eV (Fig. 2h). Consequently, the information above shows that a Ni_4_N/Ni_3_ZnC_0.7_ heterojunction was successfully synthesized.

### 3.2 Photothermal effect, enzyme-like catalytic properties of HNiZn

**Fig. 3:**
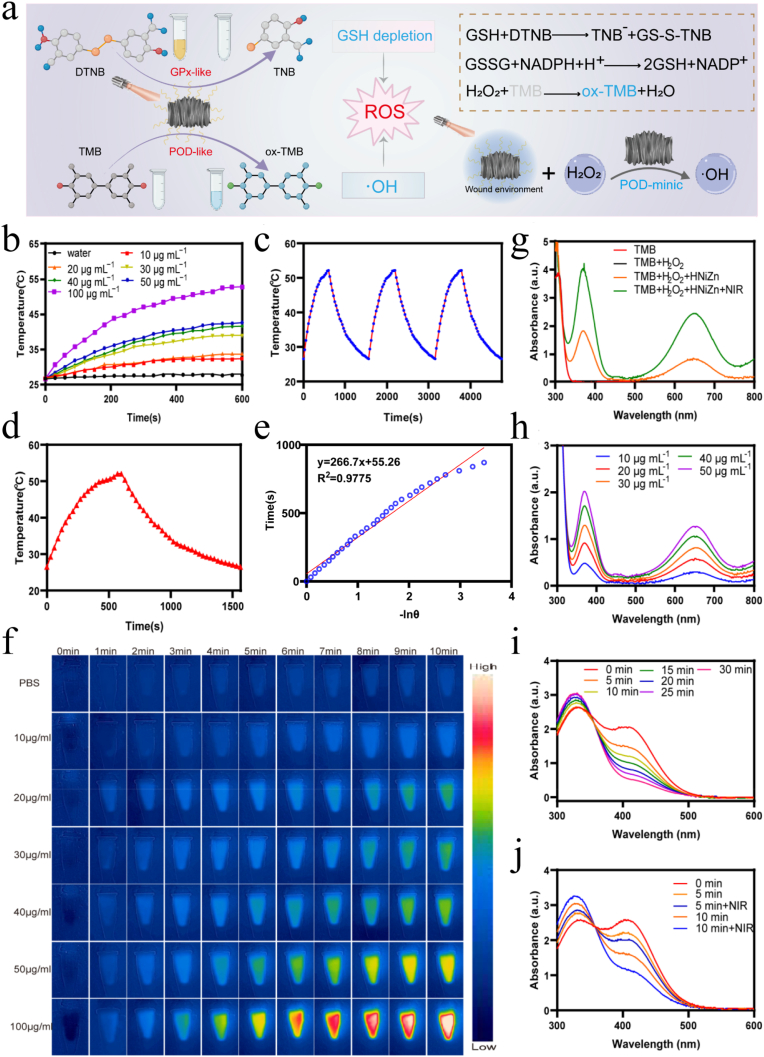
Photothermal effect, enzyme-like catalytic properties of HNiZn. (a) Schematic diagram of HNiZn generation and ·OH by TMB analysis and GSH depletion by HNiZn by DTNB analysis. (b) Warming curves of different concentrations of HNiZn under 808 nm laser irradiation (1.0 W cm^−2^, 10 min). (c) Photothermal stability (3 laser on/off cycles) of HNiZn (50 μg mL^−1^). (d) Temperature change of HNiZn (50 μg mL^−1^) for one heating and cooling. (e) Plot of cooling time versus the negative natural logarithm of the temperature drive. (f) Thermograms of different concentrations of HNiZn under 808 nm NIR laser irradiation (1.0 W cm^−2^, 10 min). (g) UV–Vis–NIR spectra of POD-like activities of HNiZn with or without 808 nm laser were examined by chromogenic TMB. (h) UV–Vis–NIR spectra of TMB of HNiZn at different concentrations (10, 20, 30, 40, 50 μg mL^−1^) in the presence of H_2_O_2_ (0.1 M). (i) UV–Vis–NIR spectra of GSH depletion at different times in the presence of HNiZn (50 μg mL^−1^). (j) UV–Vis–NIR spectra of HNiZn's ability to consume glutathione examined by colorimetric DTNB with or without the addition of 808 nm NIR laser.

Due to their ability to kill bacteria by inducing a reactive oxygen species (ROS) storm, nanozymes with POD-like and GPx-like properties are useful antimicrobial agents. PTT provides an alternative strategy to enhance the enzyme-like activity of nanozymes [27], thereby conferring them with superior antibacterial properties. Considering the depth of organizational penetration and biosafety, the PTT performance of HNiZn under 808 nm laser irradiation (1 W cm^−2^, 10 min) was systematically evaluated. The extinction coefficient of HNiZn at 808 nm was found to be 2.5 L g^−1^ cm^−1^ (Fig. s5), demonstrating its promising near-infrared (NIR) absorption characteristics. The temperature increased with the concentration of HNiZn solution, as shown in Fig. 3b–f. After a 10 min irradiation, 100 μg mL^−1^ of HNiZn reached 52.8 °C, while pure water only reached 27.7 °C. As irradiation power increased, the temperature of HNiZn (50 μg mL^−1^) temperature rose (Fig. s6). Additionally, laser switching cycles showed that repeated irradiation had no effect on the photothermal characteristics of HNiZn, indicating that the HNiZn system is photothermally stable (Fig. 3c). HNiZn (50 μg mL^−1^) has a photothermal conversion efficiency of approximately 51.01 % (Fig. 3d and e), indicating its effectiveness as a photothermal agent and establishing a foundation for future photothermal antimicrobial therapies.

The enzyme-like activity of HNiZn was further investigated, and its capacity to generate reactive oxygen species (ROS) was evaluated (Fig. 3a). POD decomposes H_2_O_2_ into hydroxyl radicals (·OH), which subsequently react with TMB to form blue compounds with absorption maxima at 652 nm. The HNiZn + TMB + H_2_O_2_ group exhibited stronger characteristic absorption peaks at 652 nm than the H_2_O_2_ + TMB group, and the blue color of the solution deepened after near-infrared irradiation (Fig. s7), with the characteristic absorption peaks being further enhanced (Fig. 3g), indicating that HNiZn possesses strong POD-like activity, which is further enhanced upon irradiation by a near-infrared laser (NIR). Electron spin resonance (ESR) analysis further confirmed the generation of ·OH by HNiZn, consistent with the previous results (Fig. s8). The POD-like activity of HNiZn was found to be dependent on both concentration and duration (Fig. 3h–s9 and s10). However, when H_2_O_2_ was not added, no distinct characteristic peak was observed at 652 nm, indicating that HNiZn without oxidase-like (OXD) activity (Fig. s11). Furthermore, the influence of pH on POD activity was investigated as the pH of the infected microenvironment decreased, with the blue color of the solution deepening (Fig. s12) and the characteristic absorption peaks increasing (Fig. s13). Additionally, Michaelis−Menten saturation curves were constructed to investigate the enzymatic kinetics of HNiZn (Fig. s14). Based on the fitted curve, the maximum reaction velocity (Vmax) and Michaelis constant (Km) of the HNiZn-catalyzed reaction were determined to be 94.72 nM s^−1^ and 0.239 mM, respectively. Compared to natural horseradish peroxidase (HRP) or other nanozymes, HNiZn had a higher Vmax and lower Km, indicating a higher affinity for the substrate and stronger POD-like activity(Tables2)**.** Bacterial infection microenvironments typically contain high levels of glutathione, which scavenges ROS to promote bacterial multiplication, mimicking GSH depletion by GPx-like and facilitating sterilization. The GSH consumption capacity was evaluated using the 5,5′-dithiobis-(2-nitrobenzoic acid) (DTNB) probe (Fig. 3a). As shown in Fig. 3i, the glutathione GPx-like activity of HNiZn was detected with GSH and DTNB as substrates, and the characteristic peaks of DTNB were largely absent after 30 min of reaction. The activity was also both time- and GSH concentration-dependent (Fig. s15), suggesting that the glutathione POD-like enzyme of HNiZn exhibits good catalytic performance and consumes GSH in a concentration-dependent manner (Fig. s16). Upon exposed to an NIR laser, HNiZn's GPx-like activity was significantly increased (Fig. 3j). To further investigate the kinetics of GSH depletion by HNiZn, revealing Vmax of 123.0 nM s^−1^ and Km of 0.571 mM (Fig. s17). As a result, HNiZn exhibits not only excellent photothermal conversion properties but also superior POD-like and GPx-like activities, which will be further enhanced by near-infrared laser (NIR) irradiation, making it a very promising antimicrobial agent.

### 3.3 DFT study on the enzymatic activity mechanism of Ni_4_N/Ni_3_ZnC_0.7_ heterojunction nanozymes

**Fig. 4:**
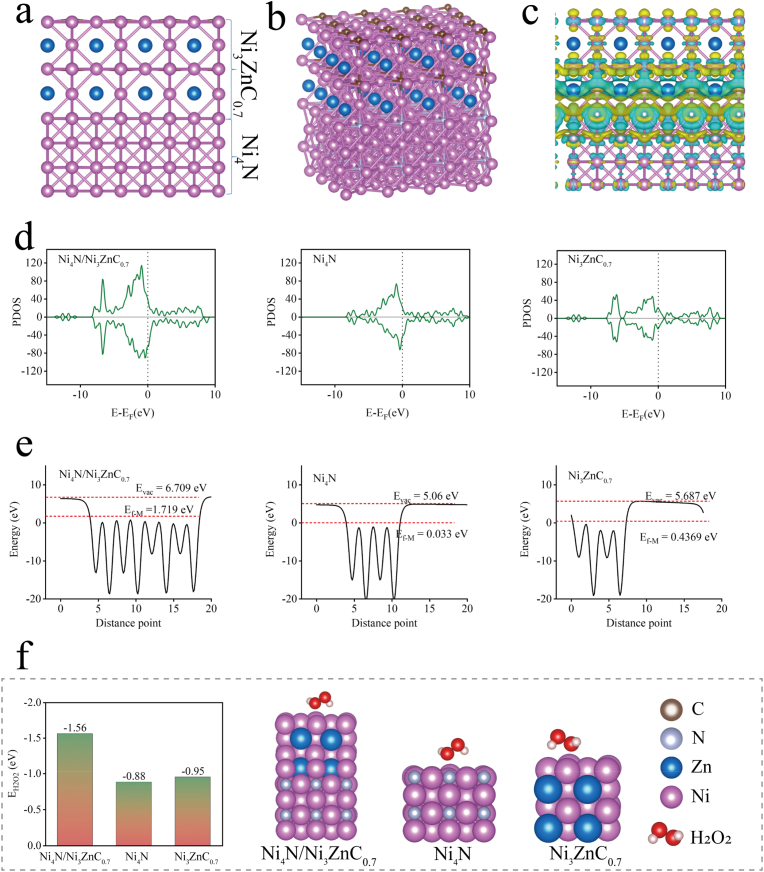
(a, b) Catalyst modelling of Ni_4_N/Ni_3_ZnC_0.7_. (c) Differential charge density profile of Ni_4_N/Ni_3_ZnC_0.7_. (d) Projected density of states (PDOS) profiles of Ni_4_N/Ni_3_ZnC_0.7_, Ni_4_N and Ni_3_ZnC_0.7_. (e) Calculated figure of merit for Ni_4_N/Ni_3_ZnC_0.7_, Ni_4_N and Ni_3_ZnC_0.7_. (f) H_2_O_2_ energy maps of Ni_4_N/Ni_3_ZnC_0.7_, Ni_4_N and Ni_3_ZnC_0.7_.

DFT calculations were performed to investigate the potential mechanism by which Ni_4_N/Ni_3_ZnC_0.7_ heterojunction nanozymes exhibit superior POD-like enzyme activities. Models were constructed based on the structural characterization of Ni_4_N/Ni_3_ZnC_0.7_ nanozymes (Fig. 4a and b). The differential charge density distribution plots showed that Ni_4_N/Ni_3_ZnC_0.7_ heterojunction nanozymes exhibited a stronger electron cloud distribution and interactions between heterogeneous atoms (Fig. 4c), which was favorable for enhancing the catalytic reaction activity of the enzyme-like enzyme. Moreover, the projected density of states (PDOS) distribution plots (Fig. 4d) showed that Ni_4_N/Ni_3_ZnC_0.7_ occupied a higher Fermi energy compared with Ni_4_N and Ni_3_ZnC_0.7_, indicating a higher electron transfer efficiency on Ni_4_N/Ni_3_ZnC_0.7_. Notably, as shown in Fig. 4e, the work function of Ni_4_N/Ni_3_ZnC_0.7_ (4.99 eV) is smaller than that of Ni_4_N (5.03 eV) and that of Ni_3_ZnC_0.7_ (5.25 eV), which is favorable for electrons escape. In particular, for Ni_4_N/Ni_3_ZnC_0.7_, these escaping electrons can more effectively enhance the enzyme-like catalytic activity under NIR illumination. Meanwhile, the H_2_O_2_ energy map (Fig. 4f) showed that the adsorption energy of H_2_O_2_ on Ni_4_N/Ni_3_ZnC_0.7_ was higher by 1.56 eV compared to Ni_4_N and Ni_3_ZnC_0.7_, indicating that the catalytic activity of Ni_4_N/Ni_3_ZnC_0.7_ was more significant for the POD reaction. Therefore, Ni_4_N/Ni_3_ZnC_0.7_ heterojunction nanozymes exhibit excellent POD-like and other catalytic activities due to their rich electron cloud distribution, stronger interactions between heterogeneous atoms, better electron transfer efficiency, lower electron escape work and stronger adsorption energies for free radicals.

### 3.4 *In vitro* antimicrobial properties and mechanism of HNiZn

**Fig. 5:**
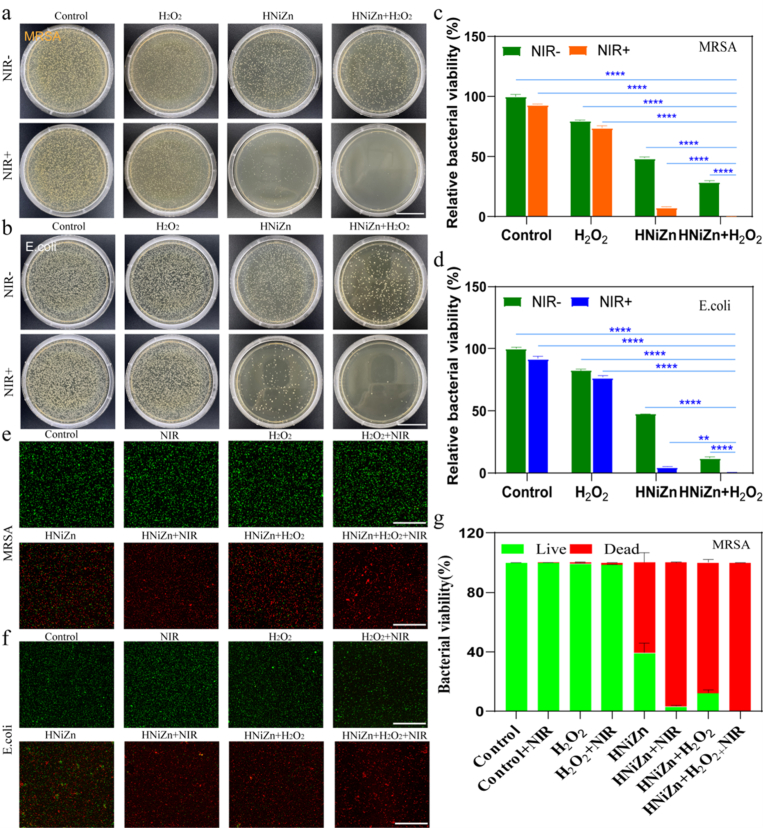
In vitro antimicrobial effect of HNiZn. Photographs of (a) MRSA and (b) *E. coli* colonies after different treatments (Control; NIR; H_2_O_2_; NIR + H_2_O_2_; HNiZn; HNiZn + NIR; HNiZn + H_2_O_2_; HNiZn + H_2_O_2_+NIR). (scale bar: 3 cm). (c)–(d) Relative bacterial viability of MRSA and *E. coli* after treatment with HNiZn according to (a)–(b). N01/PI fluorescence staining images of (e) MRSA and (f) *E. coli* after different treatments and (g) fluorescence quantification of MRSA live-dead staining. (scale bar: 100 μm). Significant differences are indicated by mean ± SD and asterisks (∗∗p < 0.05, ∗∗∗∗p < 0.0001).

The antibacterial capabilities of HNiZn were further investigated using methicillin-resistant MRSA and *E. coli* models, accounting for its better photo-heating, ROS storm formation, and glutathione consumption features. Fig. 5a and b compares the MRSA group with the Control group, demonstrating that the number of bacterial colonies treated with HNiZn and HNiZn + H_2_O_2_ decreased by 51.4 % and 71.1 %, respectively. In the *E. coli* group, the number of bacterial colonies treated with HNiZn and HNiZn + H_2_O_2_ decreased by 52.4 % and 88.1 %, respectively. After being subjected to an 808 nm NIR laser, the HNiZn and HNiZn + H_2_O_2_ groups exhibited a much lower number of colonies compared to the Control group, especially the HNiZn + H_2_O_2_ group exhibited an antibacterial rate of up to 99.9 % (Fig. 5c and d). Bacteria in each group were subjected to dead/live staining (PI and N01) for direct observation of the bactericidal effect (Fig. 5e and f) and fluorescence quantification (Fig. 5g–s18), with results comparable to previous findings. Thus, the highly effective POD-like and GPx-like activities of HNiZn provided it with strong antibacterial capacity, particularly under near-infrared light, highlighting the antimicrobial efficiency of the combination catalytic therapy of HNiZn + PTT.

**Fig. 6:**
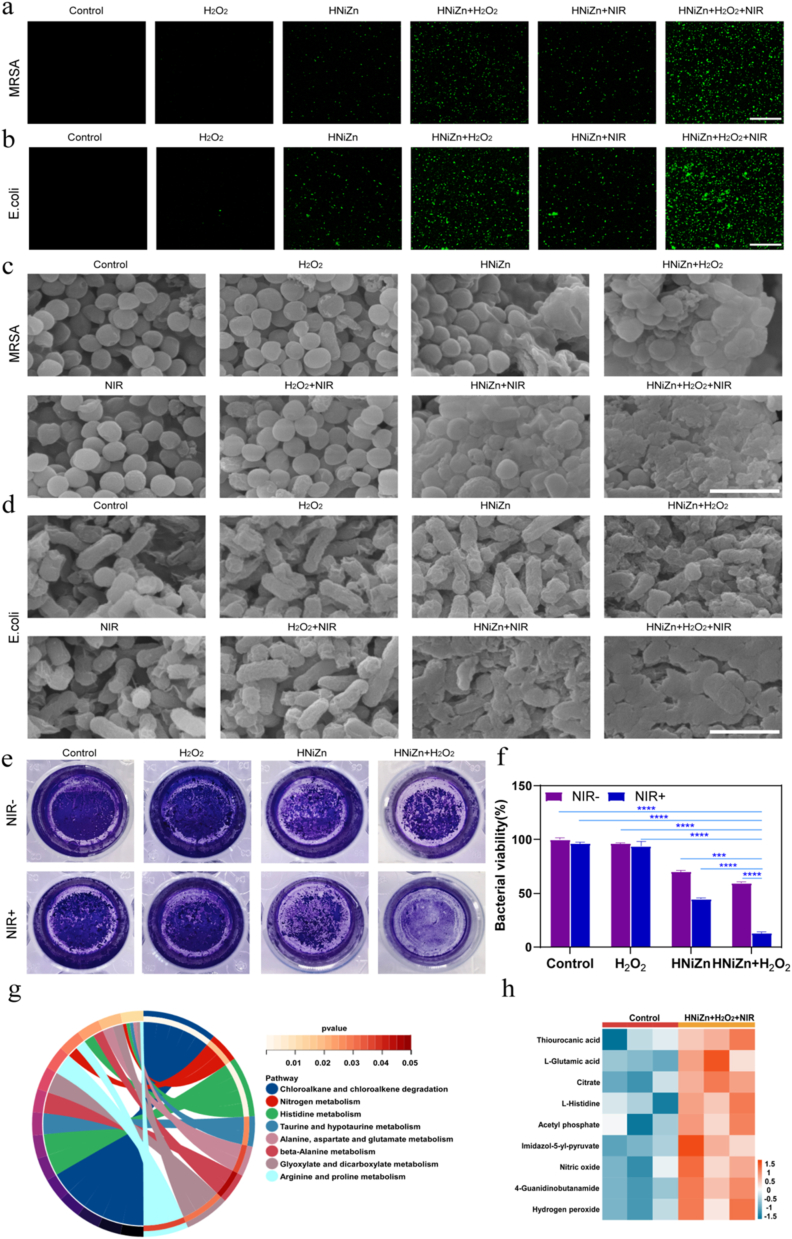
(a) Fluorescence staining images of O11 probe of MRSA and (b) *E. coli* after different treatments (Control; H_2_O_2_; HNiZn; HNiZn + H_2_O_2_; HNiZn + NIR; HNiZn + H_2_O_2_+NIR). (scale bar: 30 μm). (c) Scanning electron microscopy images of MRSA and (d) *E. coli* after different treatments (Control; NIR; H_2_O_2_; NIR + H_2_O_2_; HNiZn; HNiZn + NIR; HNiZn + H_2_O_2_; HNiZn + H_2_O_2_+NIR). (scale bar: 2 μm). (e) Crystalline violet staining of MRSA biofilms under the indicated treatments. (f) MRSA biofilm biomass was assessed on OD590 using an enzyme labeler. (g) Differences in major metabolic pathways in the HNiZn + H_2_O_2_+NIR group. (h) Heat map of the differential regulation of major metabolites in the HNiZn + H_2_O_2_+NIR group compared to the Control group. (For interpretation of the references to color in this figure legend, the reader is referred to the Web version of this article.)

The ROS level, which is closely linked to bacterial death, morphological changes in bacteria, and the impact on biofilm, was investigated in order to gain a deeper understanding of the antibacterial mechanism of HNiZn. Fig. S19 shows that the ROS levels in the bacteria progressively increased in the HNiZn, HNiZn + NIR, HNiZn + H_2_O_2_, and HNiZn + H_2_O_2_+NIR groups, compared to the Control group. It was shown that the combination of HNiZn and PTT's POD-like and GPX-like activities could produce sizable amounts of ROS in bacteria (Fig. 6a and b). Furthermore, SEM analysis of *E. coli* and methicillin-resistant MRSA after treatment with various groups showed that the primary inhibitory effect of the HNiZn and HNiZn + NIR groups was cause bacterial surface collapse (Fig. 6c and d), whereas the primary oxidative inhibitory effect of HNiZn (HNiZn + H_2_O_2_) was bacterial skeleton structure collapse. In particular, after exposure tothe NIR laser, the HNiZn + H_2_O_2_ group exhibited the greatest antibacterial effect, inflicting significant damage to practically all microorganisms. Additionally, biofilm is a multi-species bacterial community that serves as both a physical and metabolic barrier, protecting bacteria from drugs and the immune system. Therefore, the effect of HNiZn on biofilm was evaluated, as shown in Fig. 6e and f.The purple color intensity in the HNiZn + H_2_O_2_+NIR group decreased significantly by 87.3 %, indicating that HNiZn combined with PTT had a stronger biofilm eradication effect and could therefore be used as an antibacterial agent. As shown in Fig. S20, dense biofilms of viable bacteria were observed in the Control group, whereas active growth with minimal dead bacteria was observed in the NIR, H_2_O_2_, and H_2_O_2_+NIR groups. Bacterial death was increased by HNiZn treatment, due to ROS generation from its multi-enzyme activity. Compared to HNiZn, both HNiZn + H_2_O_2_ of generating toxic ·OH and HNiZn + NIR of enhancing photothermal effect demonstrated greater bactericidal effects. Notably, HNiZn + H_2_O_2_+NIR demonstrated the most potent antibacterial and biofilm-disrupting activity, which may be attributed to the photothermal and multi-enzymatic effects, consistent with previous results.

Importantly, metabolomics study of MRSA following treatment with various groups was conducted, followed by functional enrichment of these metabolites. As demonstrated in Fig. S21, the HNiZn + H_2_O_2_+NIR group exhibited higher and lower levels of 2766 and 2262 metabolites, respectively, when compared to the Control group. This was further examined using the Kyoto Encyclopedia of Genes and Genomes (KEGG). HNiZn + H_2_O_2_+NIR affected not only the metabolic pathways of histidine, aspartic acid, glutamic acid, arginine, and proline, but also the nitrogen metabolism pathway of the bacterium (Fig. 6g). In addition, key differential metabolites such as glutamate, histidine, nitric oxide, acetyl phosphate, and citrate were up-regulated in the HNiZn + H_2_O_2_+NIR group when compared to the Control group (Fig. 6h). According to the literature, carbonic anhydrase is essential for the life cycle of pathogens, and upregulation of L-glutamate inhibits bacterial carbonic anhydrase activity, interferes with bacterial pH regulation, and disrupts biosynthesis processes involving carbon dioxide or bicarbonate as substrates [28,29]; L-histidine possesses antioxidant capacity, scavenging free radicals, and the upregulation of L-histidine confirmed that this material produced a large amount of ROS [30]; Citric acid inhibits bacterial respiration, ATP synthesis, and the tricarboxylic acid cycle [31]; Nitric oxide (NO) prevents the growth of cells by causing cell cycle arrest, damaging bacterial cell membrane integrity, altering cellular function, and inducing oxidative stress [32]. Thus, these metabolites were significantly up-regulated after treatment of MRSA with the HNiZn + H_2_O_2_+NIR group, confirming that HNiZn combined with PTT treatment induces apoptosis by interfering with the bacterial biosynthesis process by affecting key enzymes required for bacterial reproduction, influencing nitrogen and energy metabolism, inducing cell cycle arrest and disrupting the bacterial redox balance.

### 3.5 *In vivo* antimicrobial action of HNiZn

To evaluate the biosafety of HNiZn, we employed the CCK-8 assay to assess the cytotoxicity of both Ni_4_N/Ni_3_ZnC_0.7_ and HNiZn on rat fibroblasts. The results showed that HNiZn significantly enhanced the survival rate of rat fibroblasts compared to the Ni_4_N/Ni_3_ZnC_0.7_ group, suggesting that HA modification effectively improves biocompatibility (Fig. s22). Furthermore, HNiZn remained stably dispersed in normal saline, phosphate-buffered saline (PBS), and DMEM α complete medium for up to 48 h (Fig. s23). Cell fluorescence staining was performed after 24 h of co-culture with or without HNiZn (Fig. s24). In HUVECs, extensive green fluorescence and minimal red fluorescence were observed, indicating high cell viability on HNiZn. And HNiZn also demonstrated excellent blood compatibility, as demonstrated by a hemolysis rate of less than 5 % at concentrations up to 100 μg mL^−1^ (Fig. s25).

**Fig. 7:**
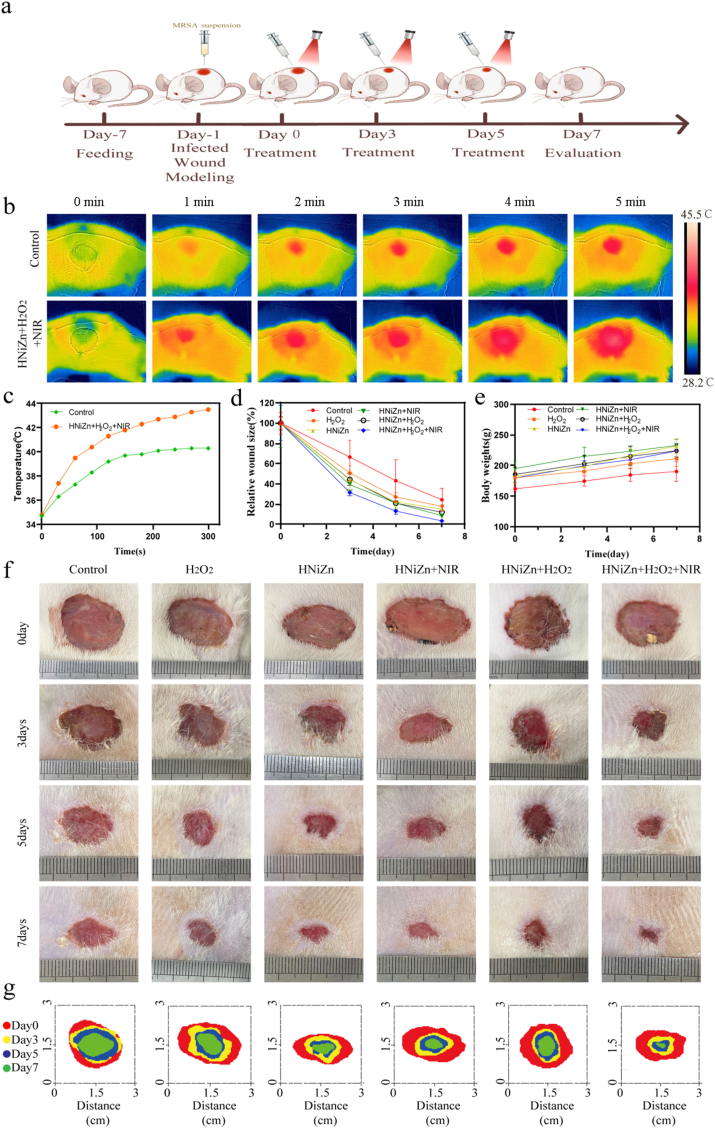
(a) Schematic diagram of MRSA infection model establishment and treatment process. (b) Thermal images of skin-infected rats before and after injection of saline or HNiZn + H_2_O_2_ and NIR irradiation for 5 min (808 nm laser, 1.0 W cm^−2^). (c) Temperature change curves of different treated rats after 808 nm laser irradiation (1.0 W cm^−2^, 5 min). (d) Ratio of decrease in wound area. (e) Weight change of rats. (f) Representative photographs of MRSA-infected wounds at 0, 3, 5 and 7 days after different treatments and (g) closure marks.

**Fig. 8:**
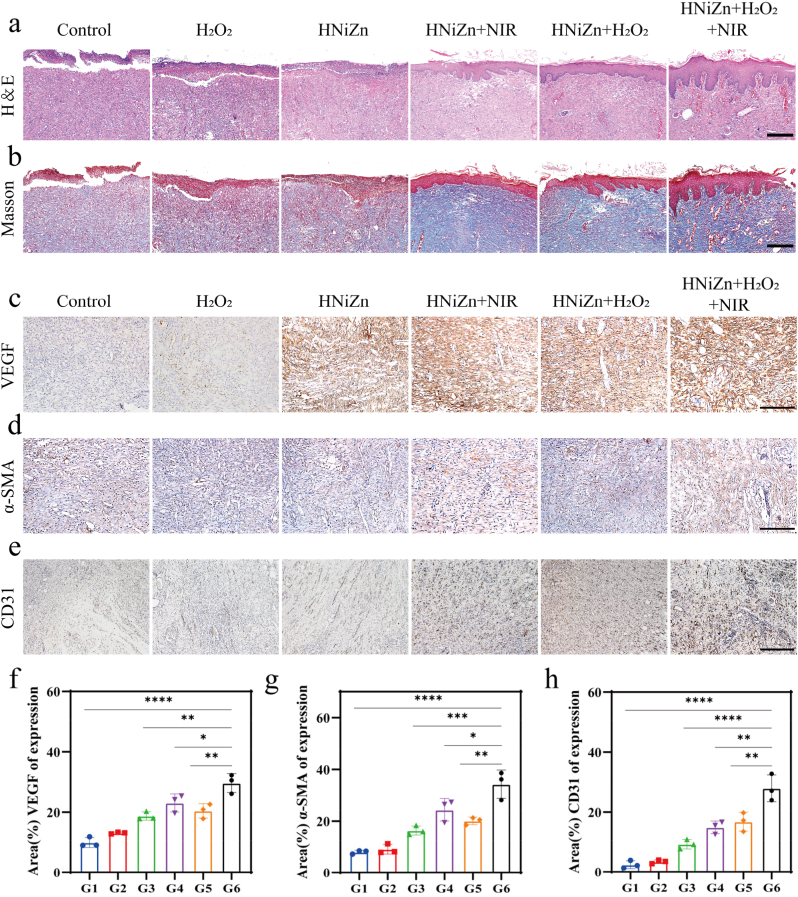
(a) HE and (b) Masson staining of bacterial infected tissues after different treatments (Control, H_2_O_2_, HNiZn, HNiZn + NIR, HNiZn + H_2_O_2_, HNiZn + H_2_O_2_ +NIR). (scale bar: 100 μm). (c) VEGF and (d) α-SMA immunohistochemistry. (scale bar: 5 μm). (e) CD31 immunohistochemistry. (scale bar: 300 μm). Relative expression levels of (f) VEGF and (g) α-SMA and (h) CD31. G1: Control, G2: H_2_O_2_, G3: HNiZn, G4: HNiZn + NIR, G5: HNiZn + H_2_O_2_, G6: HNiZn + H_2_O_2_+NIR. Data were presented as mean ± SD (n = 3). ∗*p* < 0.05, ∗∗*p* < 0.01, ∗∗∗*p* < 0.001, or ∗∗∗∗*p* < 0.0001.

As illustrated in Fig. 7a, an animal model of MRSA-infected wounds developed to evaluate the in vivo antibacterial effect of HNiZn. The HNiZn + H_2_O_2_ group exhibited a significant rise in wound temperature, reaching 43.5 °C after 5 min of 808 nm laser irradiation, compared to 40.3 °C in the control group. (Fig. 7b and c). When the temperature exceeds 50 °C, irreparable damage to the skin occurs. Therefore, the controlled wound temperature should be kept below 45 °C to achieve HNiZn combined with low-temperature PTT for wound infection treatment (Fig. 7c). Furthermore, after 7 days of therapy, the Control group exhibited a substantial tendency for wounds not to heal, indicating successful modeling (Fig. 7f). Compared to the other groups, the HNiZn + H_2_O_2_+NIR group exhibited the fastest wound area reduction and the greatest healing efficacy (Fig. 7d–f). Throughout the therapy, the body weight of rats in all groups increased steadily with no noticeable deviation (Fig. 7e). Upon completion of the wound therapy, rat wound skin was collected for hematoxylin and eosin (HE) and Masson staining to assess wound healing, as well as for immunohistochemistry staining to investigate wound neovascularization. As shown in Fig. 8a, the wounds of the HNiZn + H_2_O_2_+NIR group exhibited no substantial inflammatory reaction compared to the other groups, which manifested as skin appendages. Masson staining revealed an increased number of blue collagen fibers in the HNiZn + H_2_O_2_+NIR group in traumatized skin slices, indicating a higher density of collagen deposition compared to the other groups (Fig. 8b). Immunohistochemistry (Fig. 8c, d, e) showed that the expression of vascular endothelial growth factor, α-SMA, and CD31 was increased following HNiZn + H_2_O_2_+NIR treatment (Fig. 8f–h), indicating that light effectively stimulates angiogenesis. Therefore, the POD-like and GPx-like activities of HNiZn, combined with low-temperature PTT, successfully promote MRSA wound healing.

**Fig. 9:**
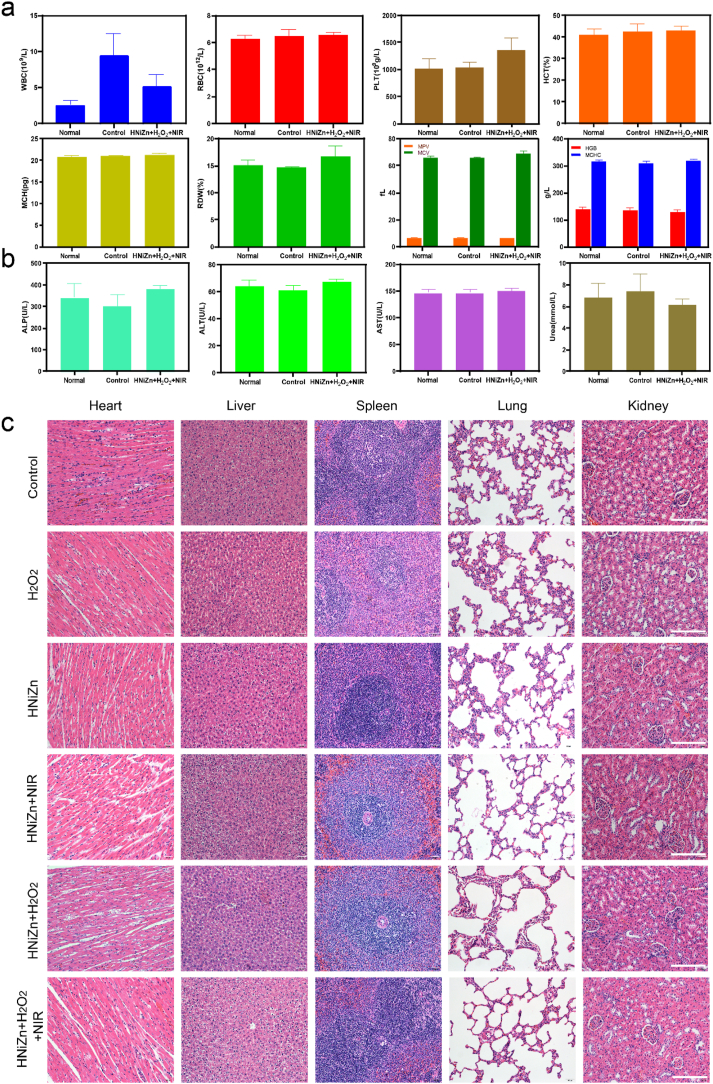
(a) Blood routine and (b) blood biochemical indexes in rats after 7 days of administration of different treatments. (c)HE staining of major organs (heart, liver, spleen, lungs, kidneys) of rats after different treatments (Control; H_2_O_2_; HNiZn; HNiZn + H_2_O_2_; HNiZn + NIR; HNiZn + H_2_O_2_+NIR), scale bar: 200 μm.

To further evaluate the biosafety of HNiZn, hemoglobin and eosin (H&E) staining was performed on the major organs (heart, liver, spleen, lungs, and kidneys) of the rats in each group after 7 days of treatment with the various methods. H&E staining revealed no significant damage, inflammation, or abnormalities in the major organs (Fig. 9c). Blood samples were obtained for routine and blood biochemistry testing (Fig. 9a and b), and the HNiZn + H_2_O_2_+NIR blood leukocytes were significantly lower than the Control group, indicating that the HNiZn + H_2_O_2_+NIR group exhibited good antibacterial properties. No significant differences were observed in the remaining blood routine and biochemistry indices between the HNiZn + H_2_O_2_+NIR group and normal healthy rats, indicating that HNiZn exhibits good biosafety and can be used as a safe and effective antimicrobial nanomaterial for photothermal enzyme catalytic therapy.

## 4 Conclusions

In summary, a unique heterostructured nanozyme of hyaluronic acid-encapsulated Ni_4_N/Ni_3_ZnC_0.7_ embedded in accordion-like nitrogen-doped carbon was innovatively synthesized via a simple molten-salt pyrolysis method. This material was applied in efficient low-temperature (43.5 °C) photocatalytic and photothermal antibacterial therapy, as well as bacterial-infected wound treatment. The heterostructure of HNiZn can generate electron holes, and had rich electron cloud distribution, strong interactions between heterogeneous atoms, low electron escape work, and strong adsorption energy for free radicals, greatly promoting electron transfer, endowing it with efficient POD-like and Gpx-like activity. Specifically, it demonstrated a high photothermal conversion efficiency (51.01 %). The photo-activated enhanced enzyme activity and photothermal effect synergistically generate abundant reactive oxygen species (ROS), which effectively kill *E. coli* and clinically isolated MRSA. Importantly, it also exhibits an effective eradication effect on the biofilm produced by MRSA. Mechanistically, metabolomics analysis revealed that HNiZn induced apoptosis by affecting carbonic anhydrase required for bacterial reproduction, interfering with bacterial biosynthesis, affecting nitrogen and energy metabolism, inducing cell cycle arrest, and disrupting bacterial redox balance. In vivo experiments demonstrated that HNiZn exhibited good biological safety, antibacterial properties, and the ability to promote collagen deposition and vascular regeneration in wound tissue, thereby facilitating skin wound healing. Therefore, this study proposes a novel strategy for the treatment of bacterial-infected wounds using heterogeneous nanozymes.

## CRediT authorship contribution statement

**Hanjie Wang:** Methodology, Investigation. **Xinqi Guo:** Methodology, Investigation. **Ying Tan:** Methodology. **Junxu Yang:** Project administration. **Yuting Ye:** Investigation. **Miao Mo:** Software. **Yanling Liang:** Software. **Guanhua Li:** Validation. **Zhangrui Huang:** Software. **Li Zheng:** Methodology. **Xiaofei Ding:** Software. **Jingping Zhong:** Visualization, Validation, Supervision. **Jinmin Zhao:** Visualization.

## Declaration of competing interest

The authors state that none of the work presented in this study may have been influenced by any known conflicting financial interests or personal ties.

## Data Availability

Data will be made available on request.
