# Supplementary material for: High electron transfer efficiency accordion-shaped HNiZn heterostructure nanozyme for low-temperature photo-catalytic enhanced therapy of bacterial infection wounds
Source: Mater Today Bio. 2025 Jul 16;34:102097. doi: 10.1016/j.mtbio.2025.102097 (PMC12305183; doi:10.1016/j.mtbio.2025.102097)
Supplement: Multimedia component 1 [file mmc1.docx]

**Supporting Information**

**High electron transfer efficiency accordion-shaped HNiZn heterostructure nanozyme for low-temperature photo-catalytic enhanced therapy of bacterial infection wounds**

Hanjie Wang,^a,b,1^Xinqi Guo,^a,c,1^ Ying Tan, ^a,1^ Junxu Yang,^a,c^ Yuting Ye,^a^ Miao Mo,^a^ Yanling Liang,^a^ Guanhua Li,^a,c^ Zhangrui Huang,^a^ Li Zheng, ^a,b, *^ Xiaofei Ding,^a,c, *^ Jingping Zhong ^a, *^，Jinmin Zhao,^a,c^

^a^ Guangxi Engineering Center in Biomedical Material for Tissue and Organ Regeneration, Collaborative Innovation Centre of Regenerative Medicine and Medical BioResource Development and Application Coconstructed By the Province and Ministry, Guangxi Key Laboratory of Regenerative Medicine, The First Affiliated Hospital of Guangxi Medical University, No. 6 Shuangyong Road, Nanning, Guangxi 530021, P. R. China

^b^ Life Sciences Institute, Guangxi Medical University, No. 22 Shuangyong Road, Nanning, Guangxi 530021, PR China

^c^ Department of Orthopaedics Trauma and Hand Surgery, The First Affiliated Hospital of Guangxi Medical University, No. 6 Shuangyong Road, Nanning, Guangxi 530021, PR China

* Corresponding author.

Correspondences to: [jingpingzhong@163.com](mailto:jingpingzhong@163.com) (J. Z), [zhengli224@163.com](mailto:zhengli224@163.com) (L. Z) and dxfeicsgk2014@163.com (X. D)

^1^The authors contributed equally to this work.

**Contents**

Supplementary Figures (Figure S1 to Figure S25)

Experimental section

Supplementary references

**Supplementary Figures (Figure S1-S25)**

**
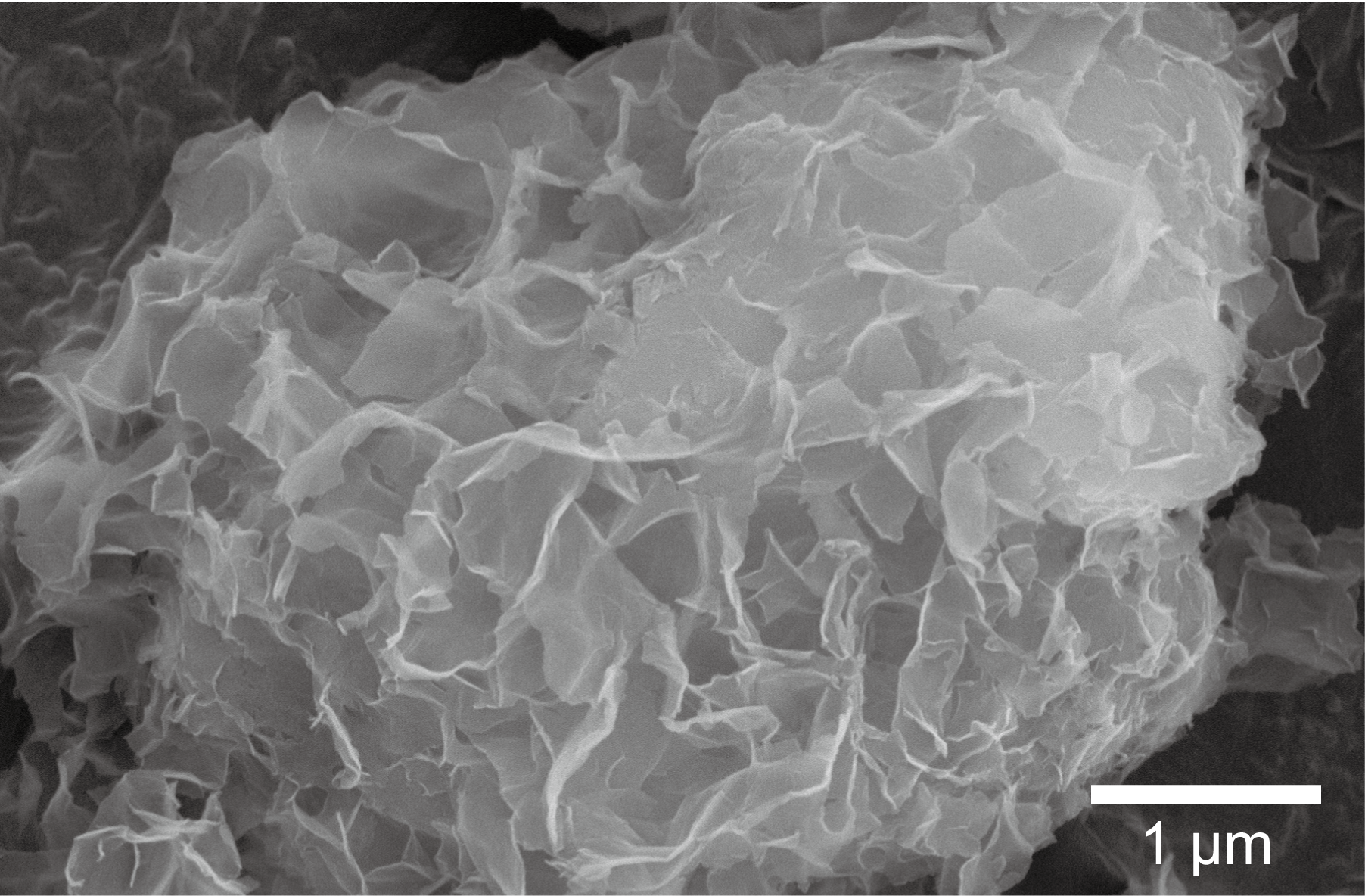
**

**Figures S1:** Scanning electron microscope image of HNiZn obtained from hyaluronic acid-modified Ni_4_N/Ni_3_ZnC_0.7_.


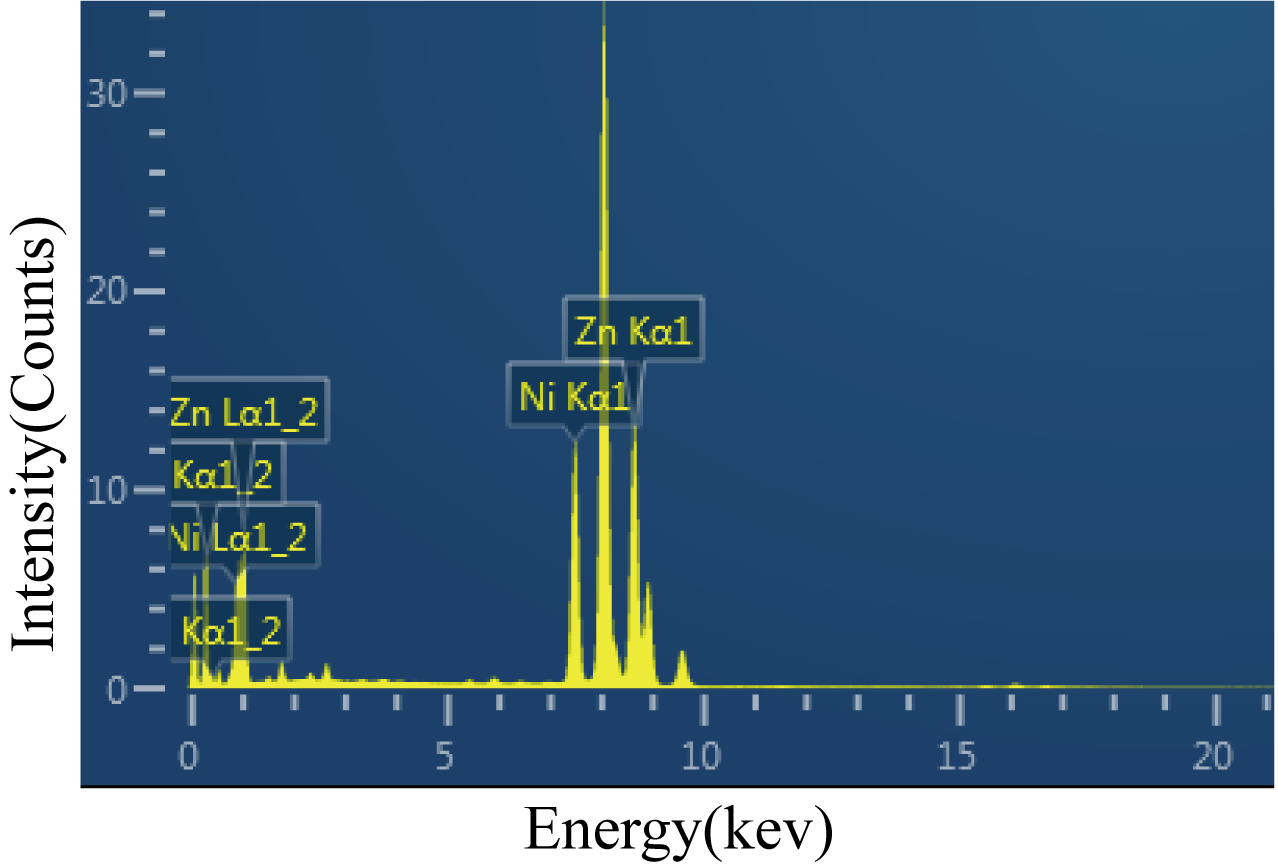


**Figures S2:** EDS images of Ni_4_N/Ni_3_ZnC_0.7_.

**Table S1:** Relative elemental content of Ni_4_N/Ni_3_ZnC_0.7_.

| Elem | wt% | wt% Sigma | Atomic percentage |
| --- | --- | --- | --- |
| C | 21.80 | 0.15 | 57.79 |
| N | 1.27 | 0.13 | 2.89 |
| Ni | 33.60 | 0.13 | 18.22 |
| Zn | 43.32 | 0.14 | 21.10 |


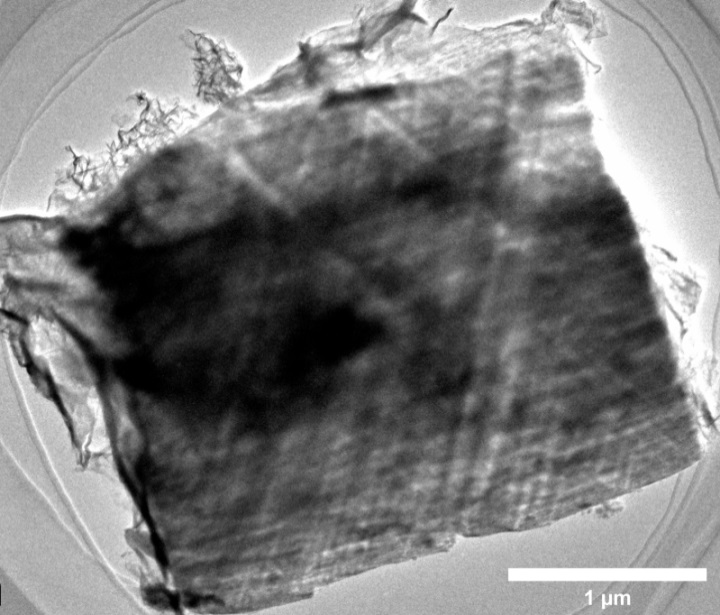


**Figures S3:** Transmission electron microscopy images of HNiZn obtained from Ni_4_N/Ni_3_ZnC_0.7_ modified by hyaluronic acid.

**Figures S4:** (a)(b) TEM images of HNiZn made from Ni_4_N/Ni_3_ZnC_0.7_ modified with hyaluronic acid; (c) High-resolution TEM (HR-TEM) images, scale bars: a is 100 nm, b is 40 nm, c is 10 nm; (d) HAADF-STEM and element mapping images of HNiZn showing C, N, O, Ni, and Zn elements, scale bar: 500 nm.

**Figures S5:** Linear fitting line between absorbance and mass concentration at 808 nm wavelength.

**Figures S6:** Temperature profiles of HNiZn solutions under 808 nm laser irradiation for 10 min at different power densities (0.5, 0.75, 1.0, and 1.5 W cm^-2^).


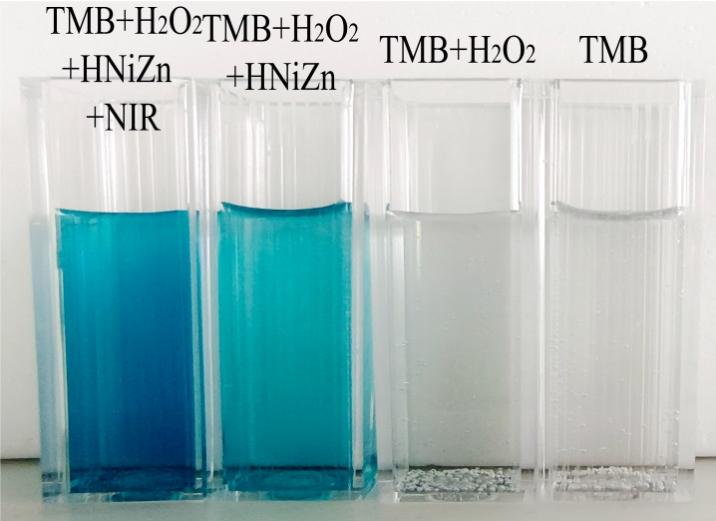


**Figures S7:** Color change of TMB under different treatment conditions at 50 μg mL^-1^HNiZn.


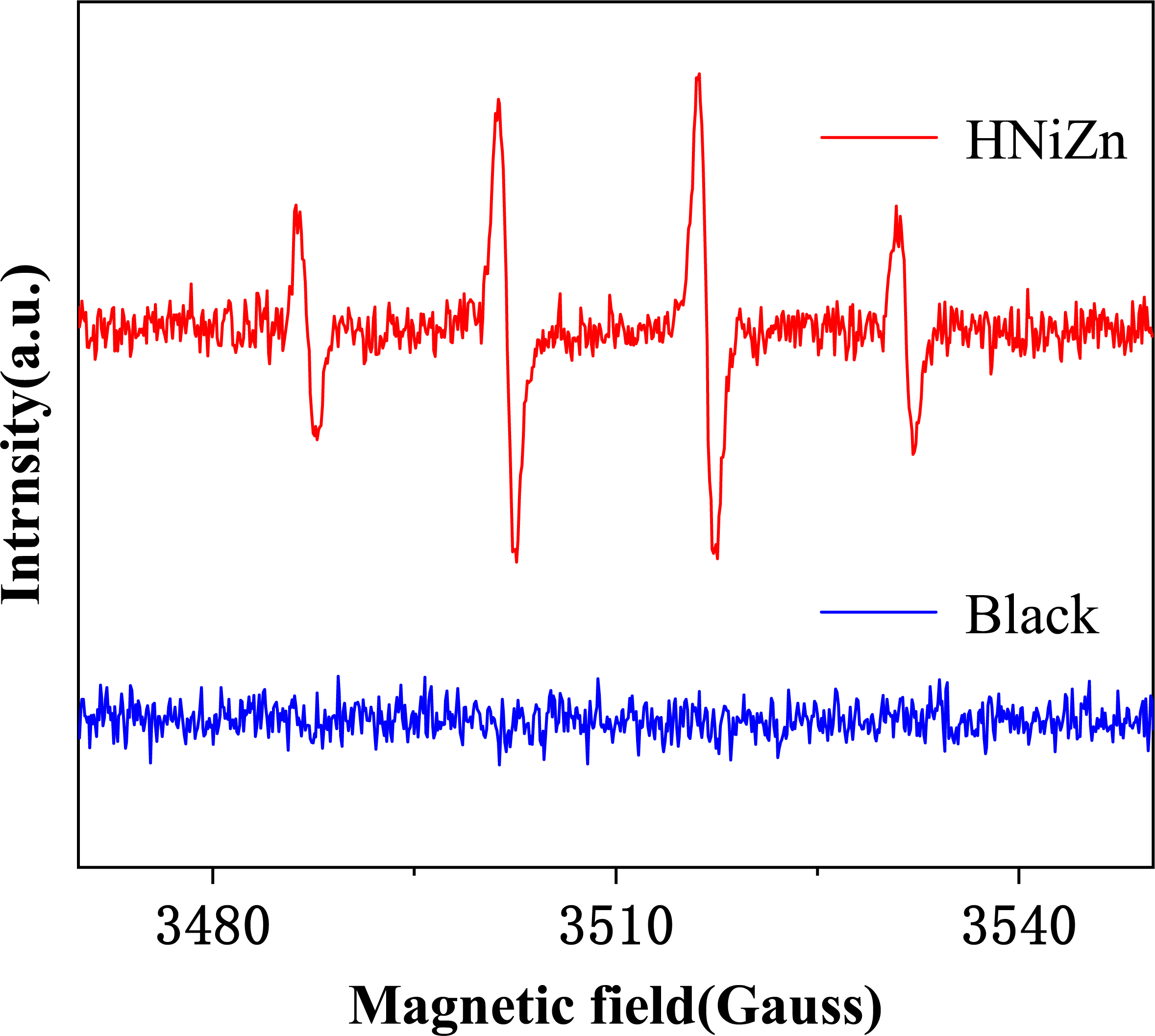


**Figures S8:** UV-visible-near-infrared spectroscopy of HNiZn+TMB color reaction under the H_2_O_2_ reaction system.

**Figures S9:** The characteristic peak change of TMB at 652 nm under 50 μg mL^-1^HNiZn at different time conditions.


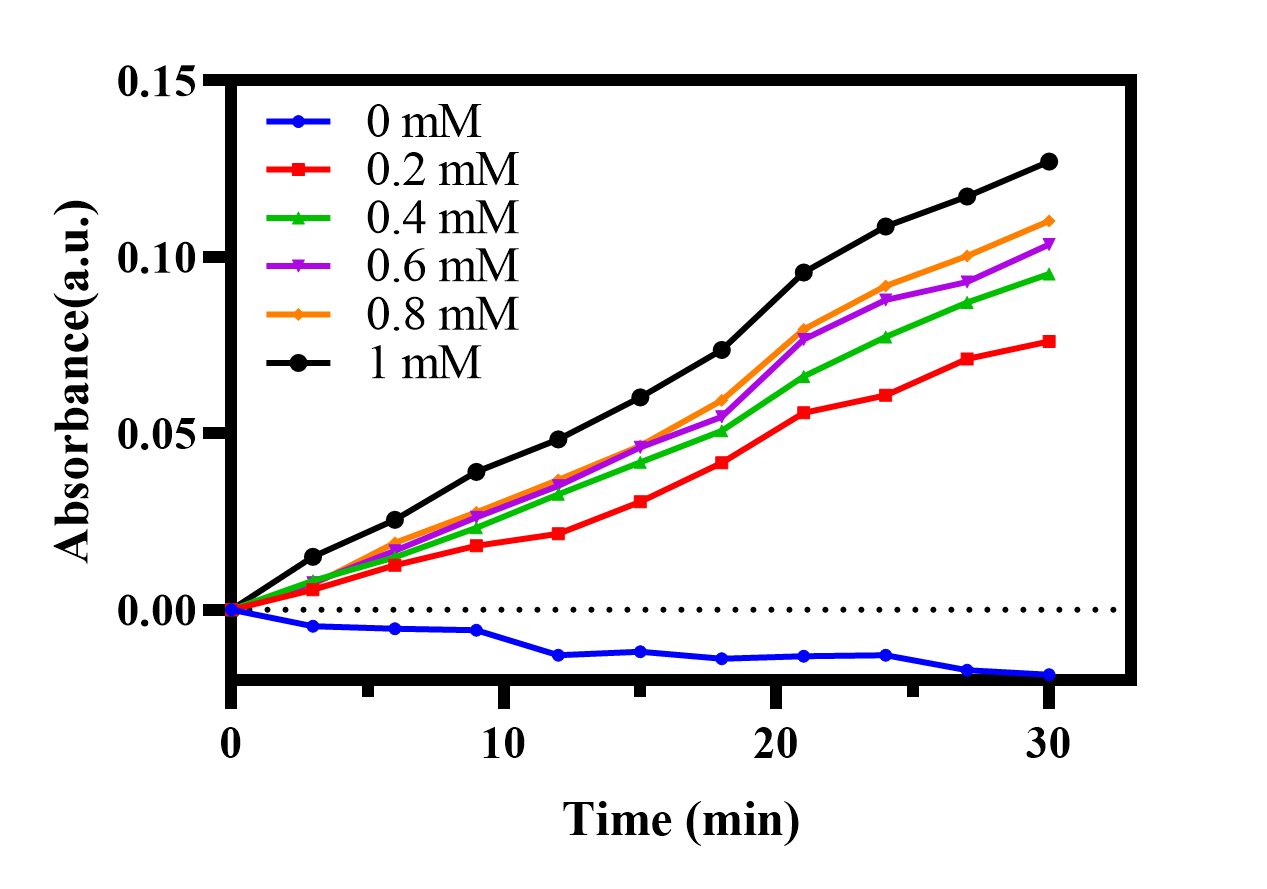


**Figures S10:** Change in absorbance of oxTMB at 652 nm by varying the concentration of hydrogen peroxide solution.


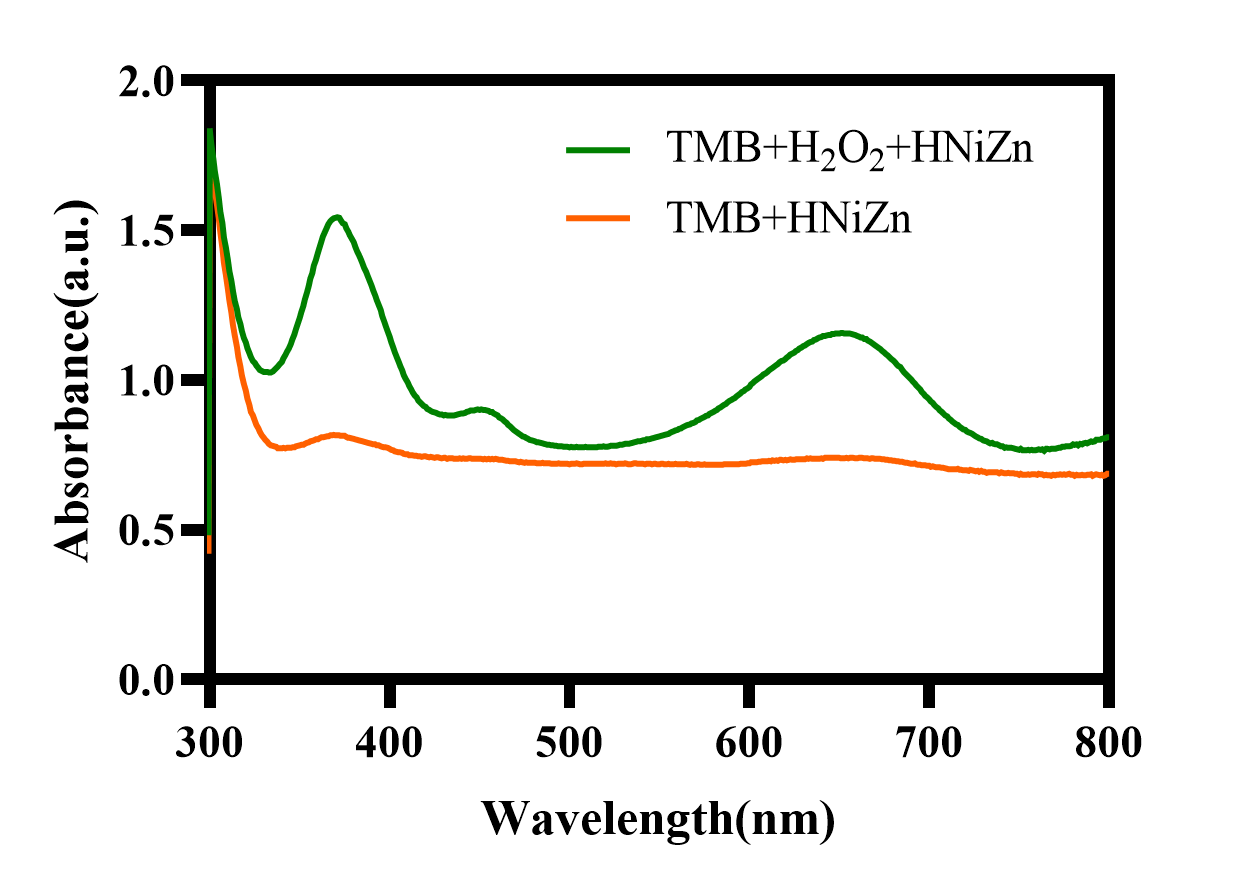


**Figures S11:** UV-visible-near-infrared spectroscopy of HNiZn+TMB color reaction under the H_2_O_2_ reaction system.


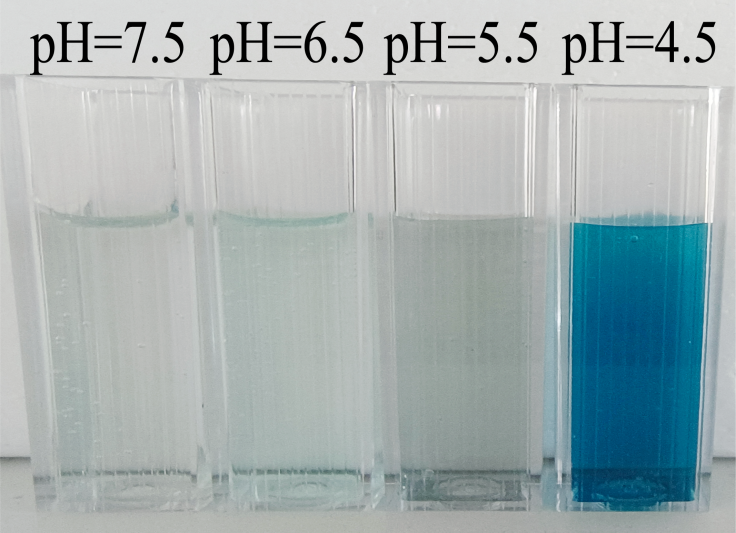


**Figures S12:** Blue color change of TMB at different pH conditions at 50 μg mL^-1^HNiZn.

**Figures S13:** The characteristic peak change of TMB at 652 nm under 50 μg mL^-1^HNiZn at different pH conditions.


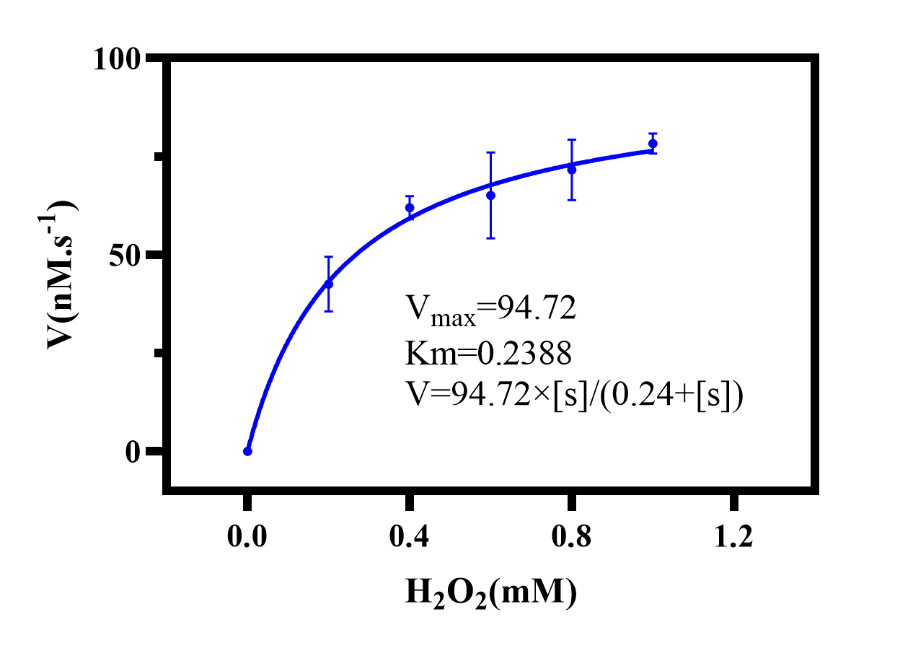


**Figures S14:** Kinetic assay of HNiZn-like peroxidase activity using hydrogen peroxide as substrate.

**Table S2:** Comparison of kinetic parameters of POD-like of HNiZn nanozyme with natural HRP and other enzymes.

| Catalyst | Km (mM) | Vmax (nM s^-1^) |
| --- | --- | --- |
| HNiZn | 0.239 | 94.7 |
| HRP | 3.7 | 87.1^[1]^ |
| Fe_3_O_4_ NPs | 150 | 0.98^[1]^ |
| Fe SACs | 0.243 | 0.0825^[2]^ |
| Fe-MOF | 0.15 | 9.2^[3]^ |
| GO | 27 | 39^[4]^ |
| IrO_2_ | 0.0764 | 4.1^[5]^ |


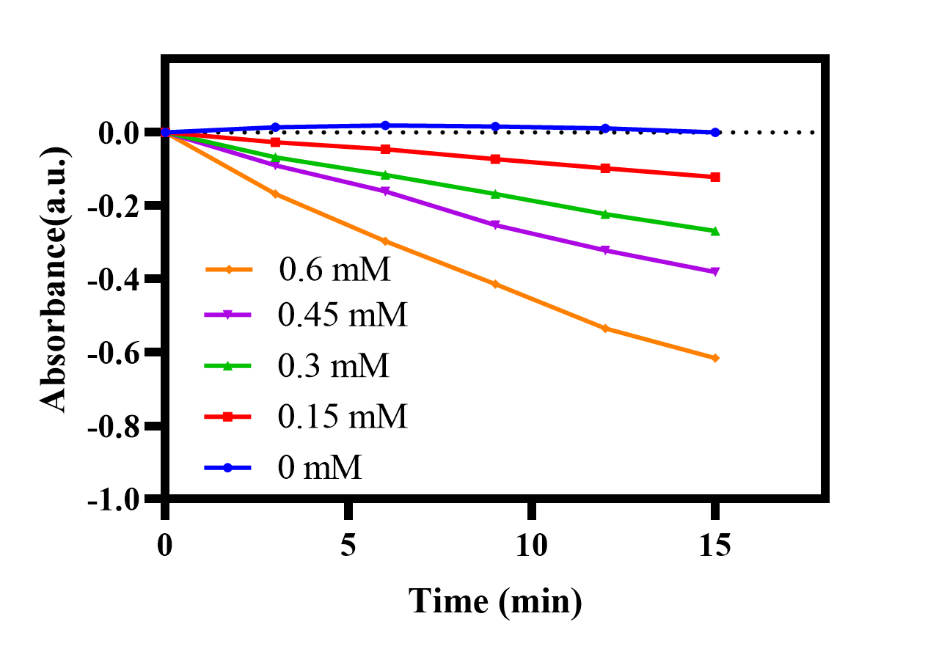


**Figures S15:** Time-dependent absorbance change of DTNB at 412 nm after 15 min treatment with different concentrations (0-0.6 mM) of GSH.

**Figures S16:** Concentration-dependent depletion of glutathione by HNiZn at the same time.

**Figures S17:** Kinetic determination of HNiZn-like glutathione peroxidase activity with glutathione as substrate.


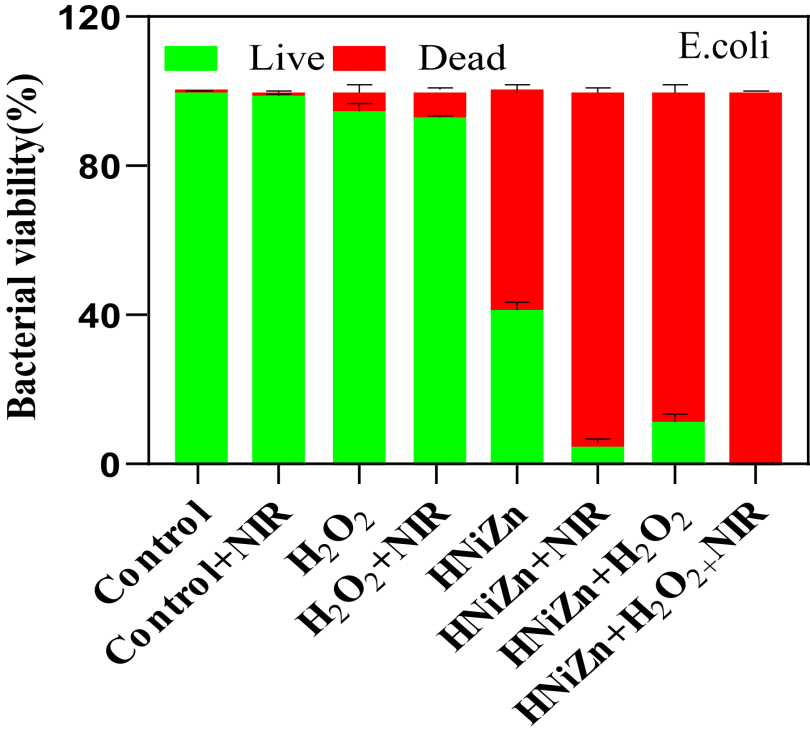


**Figures S18:** Live-dead fluorescence quantification of *Escherichia coli*.


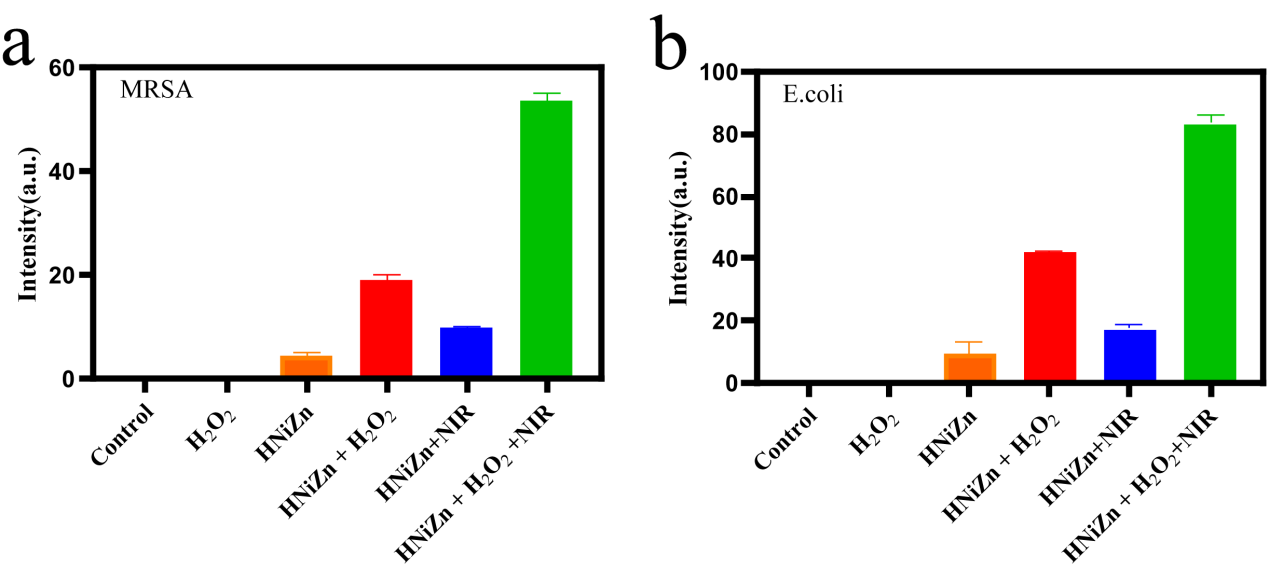


**Figures S19:** Quantification of ROS fluorescence intensity in different groups of (a) methicillin-resistant Staphylococcus aureus and (b) *Escherichia coli*.


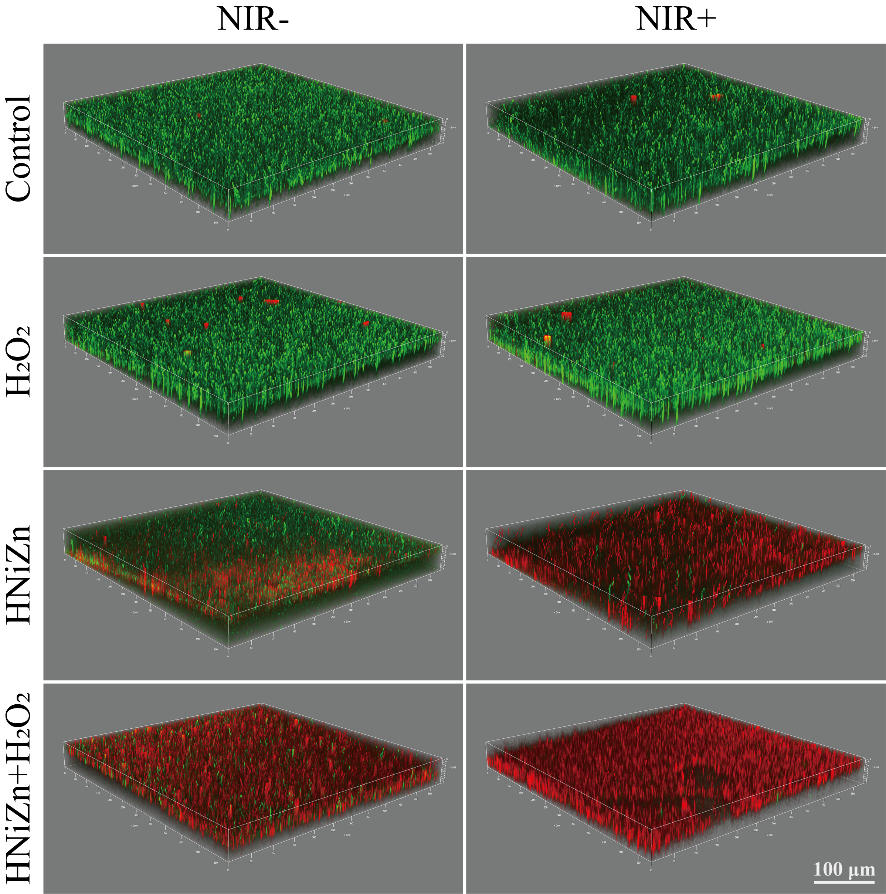


**Figures S****20:** CLSM images of MRSA biofilms stained with SYTO-9/PI under different treatments.


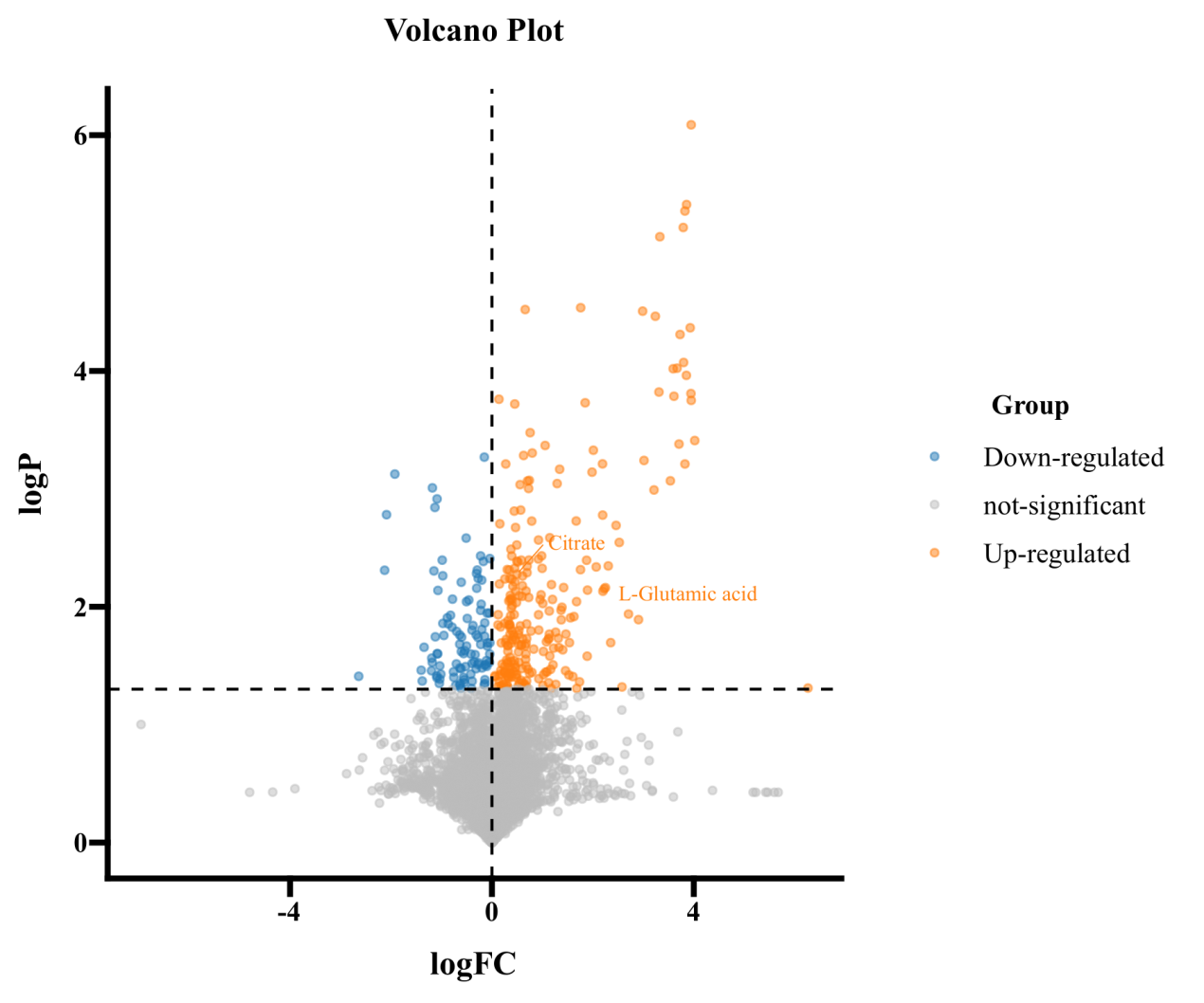


**Figures S21:** Metabolite expression levels in the HNiZn+H_2_O_2_+NIR group compared to the Control group, with yellow representing up-regulation, blue representing down-regulation, and gray representing no significance.

**Figures S22:** Relative cell viability of rat fibroblasts incubated with different concentrations of Ni_4_N/Ni_3_ZnC_0.7_ or HNiZn.

**
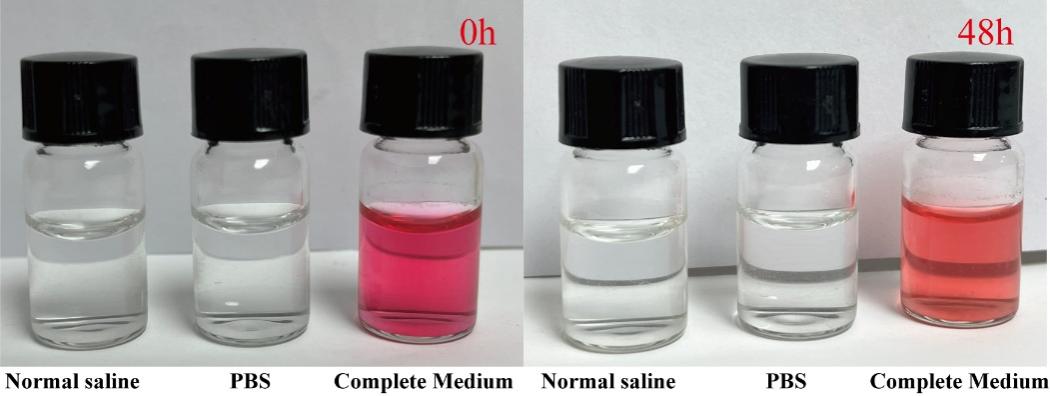
**

**Figure S23：**Dispersion stability of HNiZn in normal saline, phosphate-buffered saline (PBS), and DMEM α complete medium for 48 hours.


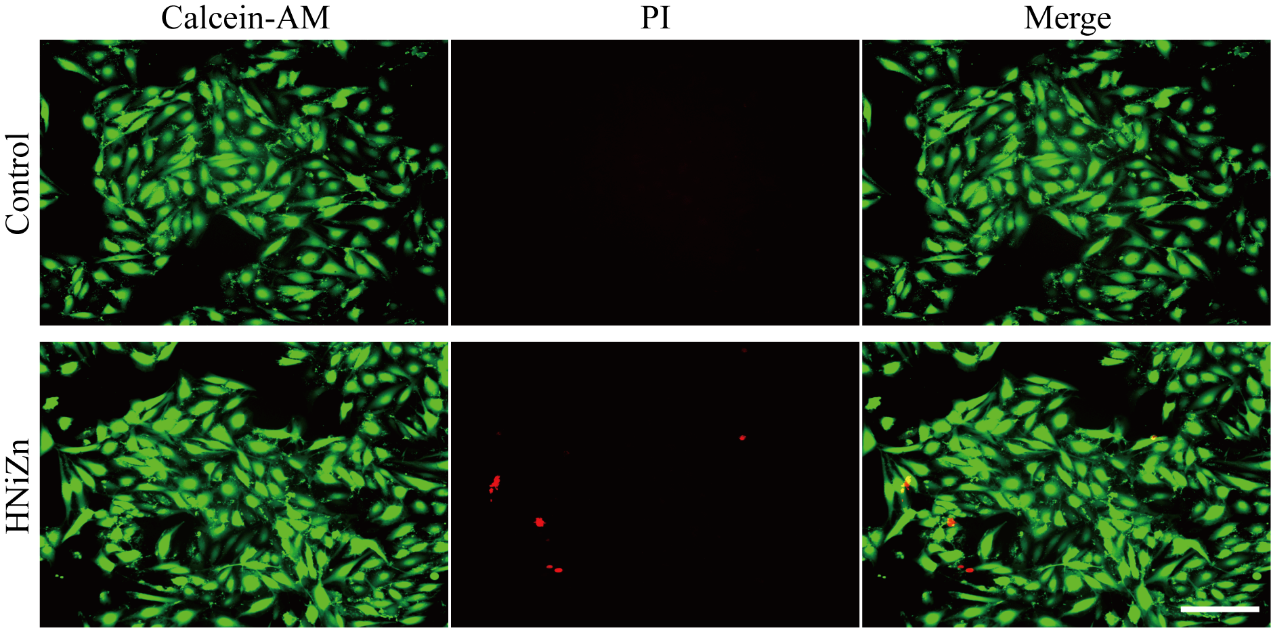


**Figures S24:** Fluorescence microscopy images of HUVECs stained with calcein AM (in green) and PI (in red) after treatment with PBS or HNiZn (50 μg mL^–1^) for 24h; scale bar: 200 μm.


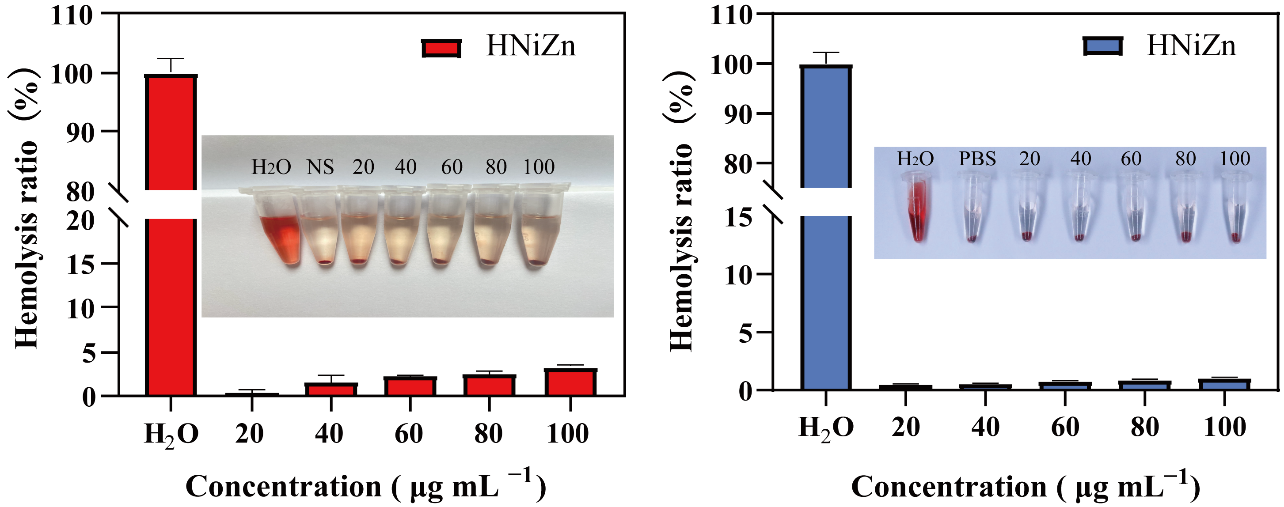


**Figures S25:** (a) Hemolysis rate of hemocytes after incubation with water and HNiZn in different concentrations of saline (b) in different concentrations of PBS solution.

**References**

[1] Gao, L.; Zhuang, J.; Nie, L.; Zhang, J.; Zhang, Y.; Gu, N.; Wang, T.; Feng, J.; Yang, D.; Perrett, S.; Yan, X., *Nature Nanotechnology* **2007**, *2* (9), 577-583. DOI 10.1038/nnano.2007.260.

[2] Zhao, C.; Xiong, C.; Liu, X.; Qiao, M.; Li, Z.; Yuan, T.; Wang, J.; Qu, Y.; Wang, X.; Zhou, F.; Xu, Q.; Wang, S.; Chen, M.; Wang, W.; Li, Y.; Yao, T.; Wu, Y.; Li, Y., *Chemical Communications* **2019,** *55* (16), 2285-2288. DOI 10.1039/c9cc00199a.

[3] Hou, D.; You, Y.; Wu, X.; Li, C.; Wu, S.; Zhang, C.; Xian, Y., *Sensors and Actuators B: Chemical* **2021,** *332*. DOI 10.1016/j.snb.2021.129508.

[4] Tu, Y.; Li, P.; Sun, J.; Jiang, J.; Dai, F.; Li, C.; Wu, Y.; Chen, L.; Shi, G.; Tan, Y.; Fang, H., *Advanced Functional Materials* **2021,** *31* (13). DOI 10.1002/adfm.202008018.

[5] Wang, D.; Zhao, S.; Li, J.; Shi, L.; Zhang, Y., *Chemical Engineering Journal* **2024,** *481*. DOI 10.1016/j.cej.2024.148888.
